# Feedforward regulatory logic controls the specification-to-differentiation transition and terminal cell fate during *Caenorhabditis elegans* endoderm development

**DOI:** 10.1242/dev.200337

**Published:** 2022-06-27

**Authors:** Chee Kiang Ewe, Erica M. Sommermann, Josh Kenchel, Sagen E. Flowers, Morris F. Maduro, Pradeep M. Joshi, Joel H. Rothman

**Affiliations:** ^1^ Department of MCD Biology and Neuroscience Research Institute, University of California Santa Barbara, Santa Barbara, CA 93106, USA; ^2^ Program in Biomolecular Science and Engineering, University of California Santa Barbara, Santa Barbara, CA 93106, USA; ^3^ Chemical and Biomolecular Engineering Department, University of California Los Angeles, Los Angeles, CA 90095, USA; ^4^ Molecular, Cell and Systems Biology Department, University of California Riverside, Riverside, CA 92521, USA

**Keywords:** Gene regulatory network, GATA factor, Cell specification, Differentiation, Endoderm, *C*. *elegans*

## Abstract

The architecture of gene regulatory networks determines the specificity and fidelity of developmental outcomes. We report that the core regulatory circuitry for endoderm development in *Caenorhabditis elegans* operates through a transcriptional cascade consisting of six sequentially expressed GATA-type factors that act in a recursive series of interlocked feedforward modules. This structure results in sequential redundancy, in which removal of a single factor or multiple alternate factors in the cascade leads to a mild or no effect on gut development, whereas elimination of any two sequential factors invariably causes a strong phenotype. The phenotypic strength is successfully predicted with a computational model based on the timing and levels of transcriptional states. We found that one factor in the middle of the cascade, END-1, which straddles the distinct events of specification and differentiation, functions in both processes. Finally, we reveal roles for key GATA factors in establishing spatial regulatory state domains by repressing other fates, thereby defining boundaries in the digestive tract. Our findings provide a paradigm that could account for the genetic redundancy observed in many developmental regulatory systems.

## Teaser

**Summary:** This study describes the architecture of the gene regulatory network underlying endoderm development in *C. elegans* and illuminates the mechanisms that provoke specification, differentiation and commitment during embryogenesis.

## INTRODUCTION

Development is driven by the progressive deployment of transcriptional programs or gene regulatory networks (GRNs), which direct specification of progenitor cells to engender different specialized cell types within a lineage and subsequent lockdown of a unique differentiated state (Boveri, 1899; Davidson and Levine, 2008; Rothman and Jarriault, 2019). This sequential restriction of cell identity and developmental potential through dynamic changes in transcriptional states was anticipated by Waddington's epigenetic landscape, a graphic metaphor describing canalization and robustness during development (Waddington, 1957).

The generation of diverse animal forms relies largely on a common genetic toolkit. GATA transcription factors (TFs) play a conserved role in the development of diverse cell types, including those of the endoderm, the first of the three germ layers to have evolved during the late Precambrian era (Hashimshony et al., 2015; Rodaway and Patient, 2001). In the diploblastic phyla Cnidarians and Poriferans, GATA factors are specifically expressed in the endoderm or in endoderm-related cells, suggesting that these transcriptional regulators may have driven the development of the endoderm germ layer and gastrulation at the dawn of metazoan evolution (Martindale et al., 2004; Nakanishi et al., 2014). This association of GATA factors with endoderm development persists throughout metazoan phylogeny, generally through successive deployment of multiple GATA factors. The sequential use of GATA factors is particularly striking in *Caenorhabditis elegans*, in which a cascade of six GATA factor-like TFs regulates specification and differentiation of the endoderm (Maduro, 2017; Maduro and Rothman, 2002; McGhee, 2007). In *Drosophila*, the GATA factor Serpent specifies endodermal fate and activates the expression of a second GATA factor, dGATAe, which is essential for the terminal differentiation of the intestine. These factors can act across wide phylogenetic spans, as first demonstrated with the *C. elegans* GATA factor END-1, which ectopically activates endoderm development when expressed in the prospective ectoderm of *Xenopus* (Shoichet et al., 2000). Similarly, overexpression of *serpent* or *dGATAe* causes ectopic endoderm differentiation in non-endodermal lineages in *Drosophila*, as well as in *Xenopus*, further supporting the functional conservation of the GATA factors (Murakami et al., 2005; Okumura et al., 2005). In sea urchin, Blimp1/Krox1 activates *otx1*, the product of which activates *gatae* expression (Davidson et al., 2002). Gatae in turn provides a positive input to *otx1*, in addition to activating the transcriptional program for endoderm development, thereby forming a stable circuit in the GRN (Davidson et al., 2002; Yuh et al., 2004). Accordingly, knocking down *gatae* severely blocks endoderm development and gastrulation (Davidson et al., 2002).

The endoderm in *C. elegans*, which arises from a single blastomere formed at the eight-cell stage known as the E cell (Boveri, 1899; Sulston et al., 1983), provides a highly tractable system for investigating the mechanisms of cell specification, differentiation and organogenesis. This progenitor cell gives rise to a clone of 20 cells comprising the intestine, which are arranged in nine rings (int1-9) spanning the length of the animal (Fig. S1A). Endoderm development is driven by three pairs of duplicated genes encoding GATA-like TFs: the divergent MED-1 and MED-2 factors (hereby referred to as MED-1/2), and the canonical END-1/3 and ELT-2/7 factors. The maternally provided SKN-1 TF (a homologue of mammalian Nrf proteins) activates MED-1 and MED-2, which initiate specification of mesendodermal fate in the EMS blastomere (Bowerman et al., 1992). In the anterior daughter of the EMS, blastomere the MS cell, the Wnt effector POP-1/Tcf represses expression of *end-1/3*, and MED-1/2 activate *tbx-35*, the product of which specifies mesodermal fate. In the E cell, which is the posterior daughter of the EMS blastomere, a triply redundant signaling system (Wnt, Src and MAPK) leads to phosphorylation of POP-1 by the nemo-like kinase LIT-1 (Bei et al., 2002; Maduro et al., 2002; Meneghini et al., 1999; Shin et al., 1999; Thorpe et al., 1997). This modified POP-1 is partially excluded from the nucleus of the E blastomere and the remaining nuclear POP-1 is converted from a repressor to an activator of endodermal fate (Maduro et al., 2005b; Shetty et al., 2005; Owraghi et al., 2010). Together with MED-1/2, Wnt-signaled POP-1 activates genes encoding the transiently expressed endoderm specification factors END-1 and END-3, which, in turn, activate the expression of ELT-7 and ELT-2, orthologues of GATA4, GATA5 and GATA6, which direct endoderm development in vertebrates (Fig. S1B). Expression of the ELT factors is sustained throughout the remainder of the life of the animal via a positive autoregulatory loop that ‘locks’ the differentiated state of the intestine (Maduro, 2017; Maduro and Rothman, 2002; McGhee, 2007). Whereas *elt-7* loss-of-function mutants do not show a discernible phenotype, animals lacking ELT-2 arrest at the earliest larval stage (L1) owing to a severely obstructed gut (Sommermann et al., 2010). Nonetheless, *elt-2(−)* animals contain a well-defined intestinal lumen and the intestinal cells appear to be fully differentiated (Fukushige et al., 1998; Sommermann et al., 2010). However, in the absence of both ELT-2 and ELT-7, the intestinal lumen is completely abolished and differentiation appears to proceed in only a subset of the endoderm-derived cells (Sommermann et al., 2010). This suggests that ELT-2 and ELT-7 function redundantly to mediate morphological differentiation of the intestine, and, in the absence of ELT-2/7, additional input(s) promote a bistable switch in the endodermal differentiation program (Dineen et al., 2018; Sommermann et al., 2010).

**Fig. 1.**
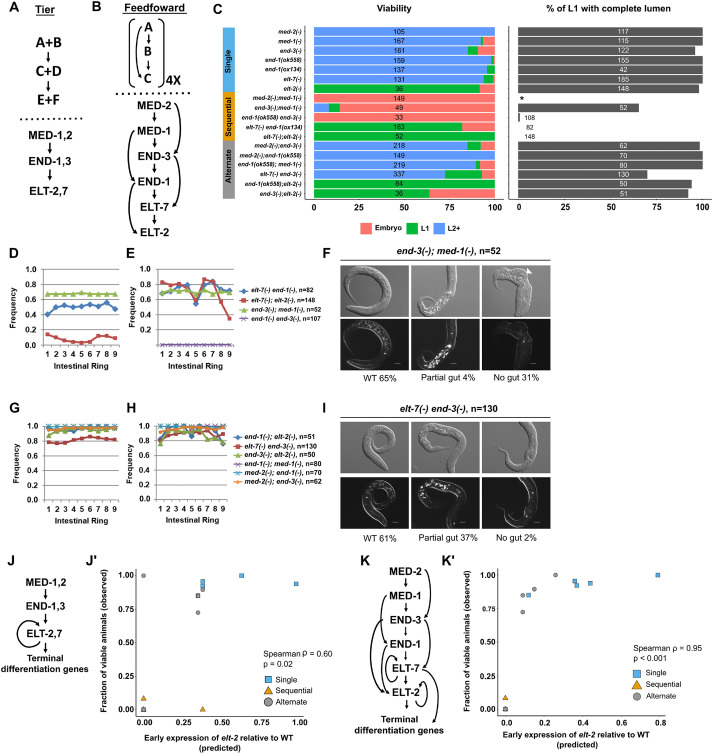
**Recursive feedforward loops in the endoderm GRN.** (A,B) Model for (A) tiered and (B) feedforward architecture in the endoderm GRN. (C) Comparison of viability and gut lumen morphology in mutant combinations lacking one or two GATA factors in the endoderm GRN. We note that the two loss-of-function alleles of *end-1*, *ox134* and *ok558* (see Table S1), do not result in significant loss in viability (Fisher's exact test; *P*=0.31) or other discernible phenotypes. We used *ox134* in subsequent analyses unless stated otherwise (see Table S3). The total number of animals scored for each genotype is indicated. No L1 animals were scored for *med-2(−);med-1(−)* (indicated by *) as all double mutants underwent embryonic arrest owing to misspecification of MS (and E in the majority of but not all embryos). (D,E,G,H) The frequencies of visible lumen (D,G) and gut granules (E,H) are dramatically reduced in double mutants lacking sequential GATA pairs (D,E) compared with those lacking alternate members (G,H) of the endoderm cascade. (F,I) *end-3(−);med-1(−)* shows a more severe defect in intestinal differentiation than *elt-7(−) end-3(−)*. The white arrowhead indicates epidermal defect in *end-3(−);med-1(−)*, resulting from the E→C transformation. WT, wild type. Scale bars: 10 μm. (J) Tiered model of the endoderm GRN with the terminal positive feedback loop between the ELTs which lock down gut differentiation. (J′) Computed *elt-2* expression levels in the tiered model. (K) The sequential feedforward model of the endoderm GRN. (K′) Computed *elt-2* expression levels in the feedforward model. All mutants show reduced *elt-2* expression relative to WT. *med-2(−);med-1(−)* double mutants show severe embryonic lethality despite *elt-2* activation owing to a fully penetrant MS→C misspecification. We excluded *med-2(−);med-1(−)* from the analyses shown in J′ and K′.

In this study, we sought to critically distinguish two alternative regulatory structures for the action of the GATA factors (paired redundant tiers versus sequential feedforward modules; Fig. 1A,B) in the endoderm GRN by two complementary approaches: genetic analysis of mutation combinations and computational modeling of the regulatory cascade. Both approaches strongly support an architecture consisting of a series of interlocking feedforward loops, creating ‘sequential redundancy’ in the cascade and culminating in the rapid lockdown of cell fate. We further report that END-1 acts at a transition point, participating in both specification and differentiation. Finally, we demonstrate the important roles of GATA factors in safeguarding intestinal cell fate and defining the boundaries of the digestive tract. Overall, our findings reveal the basis for the extensive genetic redundancy in the regulatory circuitry for endoderm development and how this GRN architecture dictates forward-driven cell specification and differentiation during embryogenesis, providing a paradigm for understanding the widespread redundancy of regulatory factors observed in many systems.

## RESULTS

The driving goal of this study was to distinguish between two alternative models for the action of the cascade of TFs underlying the endoderm GRN in *C. elegans*. Further, we sought to assess the basis for the transition between specification and differentiation, and the extent to which factors involved in the former also participate in the latter. Finally, we investigated the genetic basis underlying spatial partition of regulatory states in the digestive tract.

### Sequential redundancy suggests feedforward regulatory circuitry in the endoderm GRN

The endoderm GRN was originally interpreted as functioning through sequential pairs of redundant factors acting in three distinct tiers (two redundant MEDs acting upon two redundant ENDs, which in turn act upon two redundant ELTs) (Fig. 1A) (Maduro and Rothman, 2002). This redundancy is evident from the phenotypes of single mutants of most of the genes expressed in the *C. elegans* endoderm GRN, which show either no overt phenotype [single *med* mutants, *end-1(−)* and *elt-7(−)*] or a weakly penetrant phenotype [*end-3(−)*]. Fully penetrant loss of endoderm specification is observed only when both *med* genes or both *end* genes are removed in pairs, leading the E blastomere to adopt the fate of the C mesectodermal progenitor. Similarly, although the *elt-2(−)* mutant shows a penetrant larval arrest immediately after hatching and a dysfunctional gut, morphological differentiation of gut cells appears largely normal in these mutants; a strong gut differentiation defect requires removal of both *elt-2* and *elt-7* (Fukushige et al., 1998; Sommermann et al., 2010), indicating their redundancy in the third tier of paired redundancy.

Although these findings were consistent with the three-tiered model of redundant TF pairs, temporally resolved transcriptomic analyses revealed that the GATA factor-encoding genes are sequentially activated in the mesendoderm regulatory pathway (Fig. S2) (Baugh et al., 2003; Tintori et al., 2016). This observation is consistent with the alternative possibility that, rather than pairs of factors acting together at specific tiers in the cascade (Fig. 1A), each factor is redundant with its immediate upstream or downstream factor in a continuously sequential cascade (‘sequential redundancy’) of feedforward regulatory steps (Fig. 1B). For example, as is often seen in many similarly forward-driven biological switches, each factor may act through a feedforward motif in which a given factor activates both its immediate target gene and the target of that gene (Fig. 1B) (Mangan and Alon, 2003).

To test these two alternative models – tiered versus feedforward – we systematically analyzed a series of double mutants using null mutations of all genes and compared the penetrance of lethality and extent of gut development (Tables S1 and S3). We found that unlike the mild or undetectable phenotypes observed with single mutants, removing pairs of genes that are expressed at sequential steps in the cascade invariably resulted in severely diminished viability and gut defects. Eliminating the functions of early acting factors abrogated normal E specification, as observed in *med-2(−);med-1(−)*, *end-3(−);med-1(−)* and *end-1(−) end-3(−)* double mutants. In these cases, the endoderm precursor failed to adopt the correct identity, leading to gastrulation defects and excessive epidermis, resulting in severely disrupted embryonic morphogenesis and lethality (Goldstein and Nance, 2020). In contrast, for the ‘sequential’ double mutants that did not prevent E cell specification, as evidenced by the presence of at least some differentiated gut cells and no dramatic impact on overall embryonic morphogenesis, gut lumen morphology showed periodic interruptions, with apparently undifferentiated gaps (Fig. 1C,D). This effect was associated with sporadic expression and, in some cases, complete absence of gut-specific rhabditin granules (Clokey and Jacobson, 1986) (Fig. 1E,F). We found that expression of the gut-specific intermediate filament B-2 (IFB-2) was strongly diminished in the double mutants (Fig. S3A). Moreover, reduced apical junction molecule-1 (AJM-1) expression indicated gut epithelialization defects in the double mutants of sequential members in the endoderm GRN (Fig. S3B).

Further supporting the sequential redundancy model, the removal of two genes that function at ‘alternate’ steps in the endoderm cascade, thereby retaining an intervening factor acting between them as predicted in the feedforward model, did not result in the strong synergistic effect that we observed with the removal of pairs of sequentially expressed genes (Fig. 1C,G,H; Fig. S4). We observed normal expression of IFB-2 and AJM-1 along the length of the gut region in all such ‘alternate’ double mutants (Fig. S4). Moreover, although the *end-3(−);med-1(−)* mutants, in which sequential genes in the cascade are removed, showed severe embryonic lethality as a result of E→C misspecification, most (89.5%; *n*=219) of the *end-1(−);med-1(−)* alternate mutants developed into fertile adults (Fig. 1C). This observation is consistent with the reported divergent roles of END-1 and END-3 in endoderm specification (Boeck et al., 2011). Additionally, the alternate *elt-7(−) end-3(−)* mutants were largely viable, in contrast to the *end-3(−);med-1(−)* sequential mutants, which showed a strongly penetrant embryonic lethal phenotype (Fig. 1C). Of the hatched L1 larvae, 31% of *end-3(−);med-1(−)* sequential mutant animals exhibited no overt signs of gut differentiation at all, whereas only 2% of *elt-7(−) end-3(−)* alternate mutants completely lacked gut (Fisher's exact test; *P*<0.001) (Fig. 1F,I). However, many *elt-7(−) end-3(−)* alternate mutants contained a partially differentiated gut (Fig. 1I)*.* We suggest that the developmental defects observed in the most affected animals may result from suboptimal expression of *end-1* in the absence of *end-3* (Maduro et al., 2007; Raj et al., 2010)*.* It is striking to note that some *end-3(−);med-1(−)* animals survived to adulthood; this incomplete penetrance may be attributable to sufficient activation of *end-1* by POP-1 in a minor fraction of the embryos (Raj et al., 2010).

Although none of the single mutants showed a discernible phenotype, animals lacking both END-1 and ELT-7 were completely inviable, and the resultant arrested L1 larvae showed a striking gut differentiation defect (Fig. 1C; Fig. S3). The double-mutant worms contained patches of apparently differentiated gut, as evidenced by expression of gut granules and immunoreactive IFB-2, similar to *elt-7(−);elt-2(−)* double mutants, which exhibited an all-or-none block to differentiation event along the length of the animals (Fig. 1D,E; Fig. S3) (Sommermann et al., 2010). We note that, unlike *med-2(−);med-1(−)* and *end-1(−) end-3(−)* double mutants, in which E adopts a C fate, both *elt-7(−);elt-2(−)* and *elt-7(−) end-1(−)* mutant combinations did not exhibit any sign of cell fate transformation (highly defective embryonic morphogenesis), consistent with the failure to establish a ‘post-specification’ differentiated state in these mutants. However, the differentiation defect observed in *elt-7(−) end-1(−)* double mutants appeared to be somewhat milder than that in *elt-7(−);elt-2(−)* mutants: unlike the latter, we observed defined, albeit sporadic, lumen and brush border, and the undifferentiated patches were more frequently interspersed with differentiated patches (Fig. 1D,E; Fig. S3). This partially penetrant block to endoderm differentiation in the *elt-7(−) end-1(−)* double mutants suggests a potential role for END-3 in activating *elt-2* and gut development, as discussed below.

Analysis of the two classes of double-mutant combinations – (1) sequential and (2) alternate – revealed similar effects within, but not between each class (Fig. 1C), wherein sequential double mutants were invariably more severely affected than alternate double mutants. Together, our data support sequential redundancy throughout the cascade, reflecting a recursive series of feedforward regulatory steps. It is conceivable that such a feedforward system creates a forward-driven, rapidly deployed switch that ensures timely and robust cell fate commitment during embryogenesis.

Finally, although MED-1 and MED-2 differ by only two amino acids located outside of the DNA-binding domain and appear to perform redundant functions (Maduro et al., 2001), we were able to resolve differences in their actions, consistent with their sequential action in the cascade. In *med-2(−);med-1(−)* double mutants, all embryos died as a result of MS→C cell fate transformation (Fig. 1C), as was also the case in embryos lacking their activator, SKN-1 (Bowerman et al., 1992). In both *skn-1(−)* mutants and *med-2(−);med-1(−)* double mutants, the E cell similarly adopts a C fate in some, but not all, arrested embryos (Maduro et al., 2007). Consistent with complete redundancy, we found that removing either *med-1* or *med-2* alone did not substantially impact the expression of the endogenously tagged mNeonGreen::END-3 reporter (Fig. S5). However, our genetic analyses revealed distinguishable contributions of the two nearly identical paralogues: whereas *med-2(−);end-3(−)* double mutants showed essentially normal guts, *med-1(−);end-3(−)* double mutants showed partially defective gut development, consistent with MED-2 function preceding that of MED-1 (Fig. 1; Figs S3 and S4). Indeed, we note that, based on lineage-resolved single-cell RNA-sequencing data (Tintori et al., 2016), *med-2* transcripts are undetectable by the eight-cell stage, whereas *med-1* expression persists briefly in the endoderm precursors, suggesting that the two paralogous genes are differentially regulated.

### Computational model predicts phenotypes of sequential and alternative double mutants

We took a complementary approach to testing the two alternative GRN architectures (tiered versus feedforward) (Fig. 1A,B) through a computational strategy. We constructed a mathematical model based on the network topology, in which the interactions between the GATA factors, as well as the additional POP-1-dependent activation of *end-3* and *end-1*, were written as a series of ordinary differential equations and the model parameters were determined by fitting to published transcriptomics data (Fig. S2) (Baugh et al., 2003; Tintori et al., 2016) using a custom algorithm that followed an iterative least-squares method. We then carried out *in silico* perturbations of the two models of GRN (see Materials and Methods, and Supplementary Materials and Methods) to provide predictions of these effects on the relative timing and levels of *elt-2* activation as a proxy for the final output of the network. We found that, unlike the feedforward model, the tiered model fit poorly to the transcriptomics data (Table S4). Additionally, the computed *elt-2* expression levels in the tiered model only weakly correlated with the phenotypes of the mutant combinations (Spearman's Rank Correlation ρ=0.60; *P*=0.02) (Fig. 1J,J′). In contrast, when the feedforward model was tested, our computed results predicted that *elt-2* expression would occur with delayed onset in all single mutants and in mutants lacking ‘alternate’ pairs of GATA factors (Fig. 1K,K′); however, *elt-2* expression was predicted to be completely abrogated in double mutants in which ‘sequential’ members of the endoderm cascade were removed (Fig. 1K,K′), consistent with their pronounced developmental defects (Fig. 1C). To validate the performance of our computational model experimentally, we measured the expression of endogenously tagged mNeonGreen::ELT-2 in selected mutant combinations and compared the experimentally determined expression data to the computationally predicted *elt-2* levels (Fig. S6A-C). We found a striking and strong correlation between the measured *elt-2* expression levels and the computationally predicted values obtained from the feedforward model (Spearman's Rank Correlation ρ=1.00; *P*=0.017) (Fig. S6C). Moreover, the predicted *elt-2* expression levels strongly correlated with the experimentally observed penetrance of the phenotypes of the single and multiple mutants (Spearman's Rank Correlation ρ=0.95; *P*<0.001) (Fig. 1C,K,K′): single and alternate double mutant combinations, which showed no or weak phenotypes, were predicted to express high levels of *elt-2* early, whereas sequential double mutants, which showed strong defects in gut development and were inviable, were predicted to not express *elt-2* at substantial levels at its normal time of onset. These findings, based on computational modeling with gene expression data, coupled with experimental validation, bolster the recursive feedforward model for the GATA-factor cascade.

### Synergistic requirements and cross-regulatory interactions of END-1, ELT-7 and ELT-2

**Fig. 2.**
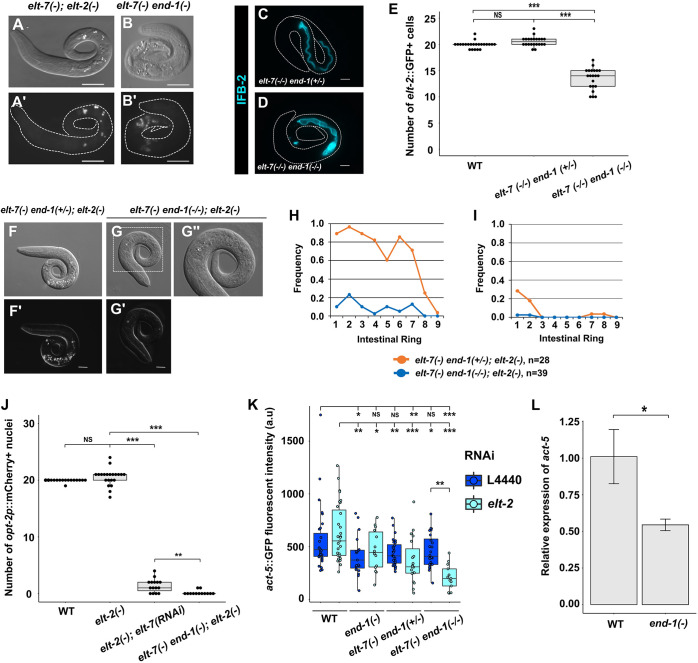
**Synergistic actions of END-1, ELT-7 and ELT-2 mediate morphological differentiation of endoderm.** (A,B) The *elt-7(*−*) end-1(*−*)* double mutant contains a defective gut with sporadic patches of rhabditin granules interspersed with apparently undifferentiated regions (*n*=82), similar to that seen in *elt-7(*−*);elt-2(*−*)* (*n*=148). Scale bars: 20 μm. (C,D) A balanced *elt-7(*−*/*−*) end-1(*−*/+)* larva shows uniform expression of IFB-2 (*kcIs6*) along the length of the animal (*n*=40), whereas the *elt-7(*−*/*−*) end-1(*−*/*−*)* larva shows sporadic expression of the transgene (*n*=42). Scale bars: 10 μm. (E) WT and *elt-7(*−*/*−*) end-1(*−*/+)* animals contain ∼20 intestinal cells, marked by the *elt-2*::*GFP* reporter. The number of differentiated gut cells is reduced in *elt-7(*−*/*−*) end-1(*−*/*−*)* (mean of 13.5 cells). (F,G) Representative micrographs of *elt-7(−/−) end-1(+/−);elt-2(−)* and *elt-7(−/−) end-1(−/−);elt-2(−)* mutants. *elt-7(−/−) end-1(+/−);elt-2(−)* animals (F,F′) lack a visible lumen and contain sporadic birefringent granules (*n*=28). *elt-7(−/−) end-1(−/−);elt-2(−)* animals (G,G′) exhibit no signs of endoderm differentiation at all (*n*=39). (G″) Magnified view of the boxed region shown in G. Scale bars: 10 μm. (H,I) The average frequencies of gut granules (H) and lumen (I) are reduced in *elt-7(−/−) end-1(−/−);elt-2(−)* compared with *elt-7(−/−) end-1(+/−);elt-2(−)*. (J) WT and *elt-2(−)* animals show similar numbers of differentiated intestinal cells, although the variance in *elt-2(−)* is significantly increased (*F*-test, *P*<0.001). The number of *opt-2p::mCherry* (*irSi24*)-expressing cells is reduced in *elt-2(−);elt-7(RNAi)* animals. No gut cells were detected in the vast majority of *elt-7(−) end-1(−);elt-2(−)* triple mutants. (K) The expression of *act-5::GFP* translational reporter *(jyIs13*) in various mutant combinations. a.u., arbitrary units. (L) *act-5* is downregulated in *end-1(−)* mutants compared to WT, as detected by RT-qPCR. *act-1* was used as the internal reference. Three replicates were performed for each genotype. Error bars represent standard deviation. For box plots, boxes represent the 25-75th percentiles and the median is indicated. The whiskers show the 1.5× interquartile range. Statistical significance was determined by non-parametric Kruskal–Wallis test and pairwise Wilcoxon tests with Benjamini–Hochberg correction (E,J), parametric one-way ANOVA followed by pairwise two-tailed unpaired *t*-tests with Benjamini–Hochberg correction (K) and two-tailed unpaired *t*-test (L). NS, not significant (*P*>0.05); *P≤0.05; **P≤0.01; ***P≤0.001.

As described above, both *elt-7(−)* and *elt-2(−)* single mutants contained a fully differentiated gut, which showed a contiguous lumen from the pharynx to the rectum and which was surrounded by cells of normal differentiated morphology. However, *elt-7(−);elt-2(−)* double mutants invariably lacked both a defined gut lumen and at least some overtly differentiated gut cells, i.e. an apparent sporadic, all-or-none, block to gut differentiation along the length of the animal (Fig. 1C-E, Fig. 2A; Figs S3 and S7) (Sommermann et al., 2010). Although differentiation was highly defective in the absence of ELT-2 and ELT-7, patches of well-differentiated gut were nonetheless evident. Moreover, many terminal differentiation genes continue to express in the absence of ELT-2 and ELT-7 (Dineen et al., 2018). For example, eliminating the functions of ELT-2 and ELT-7 had little effect on the expression of *act-5* (Fig. S8), a gene encoding an actin isoform required for microvilli formation (Dineen et al., 2018; MacQueen et al., 2005). These observations suggest that at least one additional factor, in addition to the ELTs, may activate gut differentiation. One such candidate is END-1, which acts in specification of E-cell identity immediately upstream of the *elt* genes. Indeed, although *end-1(−)* and *elt-7(−)* mutants were both phenotypically silent, we found that the *elt-7(−) end-1(−)* double mutant showed extensive gut differentiation defects, with sporadic expression of rhabditin granules (Fig. 1C-E, Fig. 2B,B′) and IFB-2 (Fig. 2C,D; Fig. S3), as well as reduced number of differentiated gut cells (Fig. 2E; Fig. S9), reminiscent of *elt-7(−);elt-2(−)* animals.

We further tested whether END-1 could account for the residual gut-promoting activity by removing it from *elt-7(−);elt-2(−)* animals. Strikingly, simultaneously eliminating END-1, ELT-7 and ELT-2 resulted in apparent abolishment of intestinal differentiation (Fig. 2F-I). We found that 19.6% of *elt-7(−) end-1(−);elt-2(−)* triple mutants underwent embryonic arrest and the remainder died as L1 larvae (*n*=143). These animals exhibited no morphological signs of gut differentiation, such as gut granules (Fig. 2F-H; Fig. S10A,C), no detectable intestinal brush border or lumen (Fig. 2F-I; Fig. S10) and no cells expressing the gut-specific peptidase transporter OPT-2 (Fig. 2J; Fig. S11), suggesting a total block to gut differentiation. We found that knocking out *end-1* strongly reduced the expression of *act-5* (Fig. 2K,L) and *act-5* expression was further downregulated in *elt-7(−) end-1(−)* and in *elt-7(−) end-1(−);elt-2(RNAi)* animals (Fig. 2K; Fig. S12), suggesting that END-1, ELT-7 and ELT-2 act collaboratively and redundantly to mediate *act-5* expression, and that END-1 may compensate for the loss of ELT-2 and ELT-7 inputs.

To investigate whether END-3, like END-1, promotes gut morphological differentiation, we knocked down *elt-7* in *end-3(−);elt-2(−)* double mutants and found that the arrested animals showed profound gut differentiation defects, as we observed in *end-1(−);elt-2(−);elt-7(−)* triple mutants (Fig. 2F-I; Fig. S10). Moreover, *act-5* was strongly downregulated in *end-3(−)* mutants (Fig. S13). Although these results may implicate a role of END-3 in driving differentiation, we cannot rule out that these effects were mediated primarily through END-1, as END-3 acts upstream to activate END-1 and *end-1* expression is downregulated in the absence of END-3 (Maduro et al., 2007; Raj et al., 2010). For this reason, we hereafter focused specifically on END-1 function in the specification-to-differentiation transition.

**Fig. 3.**
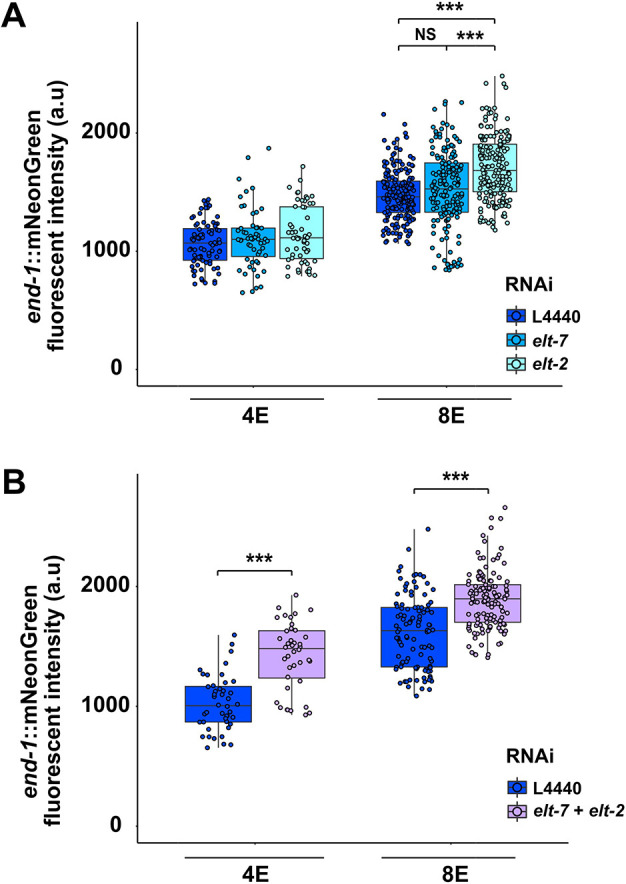
**ELT-2 antagonizes *end-1* expression.** (A) Expression of endogenously tagged *end-1* increases in 8E embryos upon *elt-2* RNAi treatment. (B) Knocking down both *elt-7* and *elt-2* further elevates END-1 expression in both 4E and 8E embryos. Boxes represent the 25-75th percentiles and the median is indicated. The whiskers show the 1.5× interquartile range. a.u., arbitrary units. Statistical significance was determined by the Kruskal–Wallis test and pairwise Wilcoxon tests with Benjamini–Hochberg correction (A) and Wilcoxon tests (B). NS, not significant (*P*>0.05); ***P≤0.001.

The notion for a role of END-1 as the gut-promoting factor acting in the absence of ELTs is challenged by the finding that its expression in wild-type embryos is transient to the degree that the protein is largely undetectable by the 16E embryonic stage (Li et al., 2019; Zhu et al., 1997); nonetheless, gut differentiation seen in the differentiated patches in *elt-7(−);elt-2(−)* mutants appears strong and robust late in development. These findings led us to hypothesize that ELT-2 and/or ELT-7 may normally repress *end-1* expression through negative feedback and that, in the absence of the ELTs, *end-1* may be upregulated and drive differentiation. Indeed, our preliminary results revealed that the expression of the *end-1* endogenous protein fused to a reporter was modestly elevated in 8E embryos when *elt-2* was knocked down by RNAi (Fig. 3A). Moreover, the expression levels of *end-1* were upregulated in both 4E and 8E embryos when ELT-7 and ELT-2 were simultaneously depleted (Fig. 3B). Although these findings provide initial evidence for the hypothesized feedback inhibition, the effect was rather weak, as we did not observe an obvious longer-term perdurance of *end-1* expression.

Taken together, our results suggest that END-1 (and possibly END-3) is poised at the interface between specification and differentiation in the cascade, placing it at the crux of this major developmental transition. END-1, acting with END-3, regulates specification of the E lineage, whereas END-1, acting with ELT-7 and ELT-2, controls differentiation of the intestine.

### ELT-2 and ELT-7 collaborate to safeguard intestinal cell fate

**Fig. 4.**
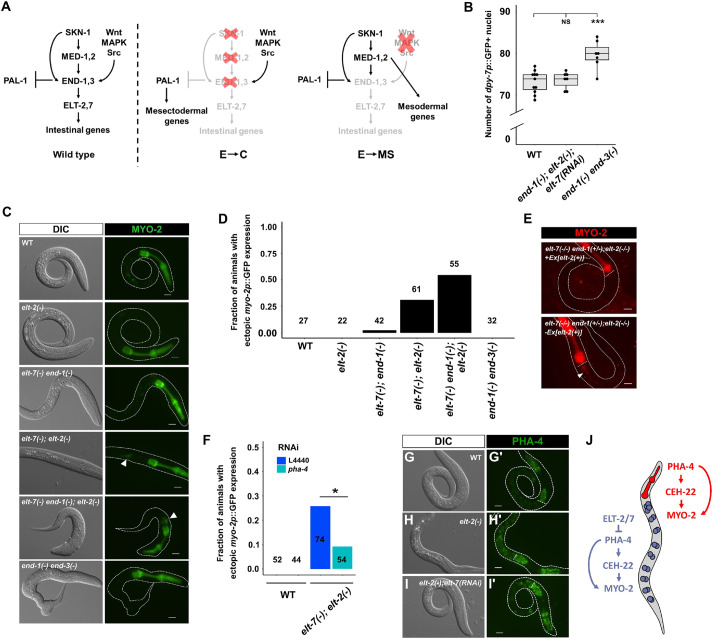
**ELT-2 and ELT-7 repress pharyngeal gene expression in the intestine.** (A) E→C or E→MS binary fate choice during early embryogenesis. PAL-1 is required for the specification of the C blastomere, which gives rise to epidermis and body wall muscles (Hunter and Kenyon, 1996). Maternally provided *pal-1* is specifically translated in EMS and P_2_. In MS and E, PAL-1 activity is blocked by TBX-35 and END-1/3, respectively (Broitman-Maduro et al., 2006). Thus, depleting TBX-35, END-1/3 or their upstream activators, MED-1/2 and SKN-1, causes excess skin and muscle owing to the misspecification of MS and/or E into C, the somatic descendant of P_2_ (Hunter and Kenyon, 1996). As EMS divides, Wnt/MAPK/Src signaling from P_2_ polarizes EMS, leading to the phosphorylation of POP-1 and activation of *end-1/3* in E, but not in MS. When the polarizing signal from P_2_ is disrupted, POP-1 is unphosphorylated and MED-1/2 instead activate the development of MS, which gives rise to the posterior pharynx and body wall muscles (Maduro and Rothman, 2002; Maduro et al., 2002; Rocheleau et al., 1999; Shin et al., 1999). (B) Both WT and *end-1(*−*);elt-2(*−*);elt-7(RNAi)* larvae contain ∼73 epidermal cells, whereas *end-1(*−*) end-3(*−*)* larvae contain ∼80 epidermal cells marked by *dpy-7p::GFP*. Boxes represent the 25-75th percentiles and the median is indicated. The whiskers show the 1.5× interquartile range. NS, not significant (*P*>0.05); ***P≤0.001 (parametric one-way ANOVA followed by pairwise two-tailed unpaired *t*-tests with Benjamini–Hochberg correction). (C) Representative differential interference contrast (DIC) and fluorescence micrographs showing different mutation combinations expressing *myo-2p::GFP*. Arrowheads indicate ectopic expression of *myo-2* in *elt-7(*−*);elt-2(*−*)* and *elt-7(*−*) end-1(*−*);elt-2(*−*)*. (D) The frequency of animals showing mis-expression of *myo-2*, as shown in C. The number of animals scored for each genotype is indicated. (E) Fluorescence micrographs showing expression of *myo-2p::mCherry* present on *tmC12*, which balances *end-1(*−*)*. An *elt-7(*−*/*−*) end-1(+/−);elt-2(*−*/*−*)* animal that has lost the *elt-2(+)* rescuing array shows ectopic expression of *myo-2* in the midgut (arrowhead) (*n*=50). The white horizontal lines mark the posterior end of the pharynx. (F) Knocking down *pha-4* partially rescues ectopic expression of *myo-2* in *elt-7(*−*);elt-2(*−*)* animals. *P≤0.05 (Fisher's exact test). (G-I) The expression of the endogenously tagged *pha-4* reporter in WT (G,G′) (*n*=120), *elt-2(*−*)* (H,H′) (*n*=40) and *elt-2(*−*);elt-7(RNAi)* (I,I′) (*n*=30) animals. The white horizontal lines in G′-I′ mark the posterior end of the pharynx. Exposure time: 195 ms. All scale bars: 10 μm. (J) Model of spatial repression and fate exclusion in the digestive tract.

Given that END-1 straddles the transition from specification of the endoderm progenitor and differentiation of the gut, we sought to investigate whether specification and differentiation involve distinct regulatory events. To do so, we examined non-endodermal (epidermal and pharyngeal) gene activity when differentiation was impaired at a point after this transition was thought to occur. When improperly specified, E undergoes extensive lineage conversion to either a C-like mesectodermal fate (when SKN-1, MED-1/2 or END-1/3 are removed), thereby improperly generating epidermis, or an MS-like mesodermal fate (for example, in the absence of Wnt signaling), thereby improperly giving rise to pharyngeal tissue (details described in Fig. 4A). In *end-1(−) end-3(−)* double mutants, misspecification of the E into a C-like cell caused severe morphological defects as a result of the supernumerary epidermal cells and consequent deformation of the epidermis (Fig. 4B; Fig. S14) (Maduro et al., 2005a). In contrast, the *elt-7(−) end-1(−);elt-2(−)* triple mutant animals, which, like the *end-1(−) end-3(−)* double mutants, do not form a discernible gut, were nonetheless not substantially defective for overall body morphogenesis (Fig. S14), implying that E was not mis-specified. Consistent with this observation, unlike the *end-1(−) end-3(−)* double mutant animals, *end-1(−);elt-2(−);elt-7(RNAi)* larvae contained a wild-type number of epidermal cells, further supporting the view that E→C misspecification did not occur (Fig. 4B). These results demonstrate that END-3 alone was sufficient to promote endoderm specification over C-like mesectodermal cell fate.

Although the foregoing results suggest that the E lineage was not mis-specified when multiple late components in the cascade were eliminated, we were surprised to observe mis-expression of the pharyngeal muscle-specific myosin gene, *myo-2*, in the gut region of many *elt-7(−);elt-2(−)* and *elt-7(−) end-1(−);elt-2(−)* mutants (Fig. 4C,D). We further confirmed this observation using a different *myo-2* transgenic reporter (Fig. 4E). Additionally, we found that knocking down *elt-7* in *end-1(−);elt-2(−)* mutants caused ectopic expression of *ceh-22*, which encodes a pharynx-specific NK-2-type homeodomain protein, in the otherwise undifferentiated gut (Fig. S15). We reasoned that this inappropriate expression of pharyngeal genes was unlikely to result from an extensive E→MS transformation but may reflect later errors in the fidelity of differentiation for the following reasons: (1) Wnt/MAPK/Src signaling was unperturbed in *elt-7(−);elt-2(−)* and *elt-7(−) end-1(−);elt-2(−)*, such that the polarization of E and MS in the early embryo was expected to proceed as normal, and (2) the E→MS transformation in cell fate invariably led to profound embryonic lethality as the endodermal progenitors failed to migrate properly during gastrulation, resulting in a highly defective morphogenesis (see, for example, the ‘Mom’ phenotype in Thorpe et al., 1997). We did not observe such a phenotype in either *elt-7(−);elt-2(−)* double mutants or *elt-7(−) end-1(−);elt-2(−)* triple mutants. Finally, we found that *elt-7(−);elt-2(−)* animals contained an average of ∼14 cells expressing the *elt-2* transcriptional reporter, indicating initiation of the endoderm developmental program (Fig. S7), and a small number of these cells expressed a late gut marker (the *opt-2* reporter) (Fig. 2J; Fig. S11). Collectively, these results suggest that in *elt-7(−);elt-2(−)* animals, E and its descendants are initially specified, but fail to maintain their proper terminally differentiated fate following specification, resulting in sporadic ‘transdifferentiation’ of some of the cells.

PHA-4/FoxA is the organ selector gene that specifies pharyngeal identity, regulating *myo-2* and *ceh-22* among thousands of other targets in the pharynx (Mango et al., 1994; Zhong et al., 2010). We found that the ectopic expression of *myo-2* in *elt-7(−);elt-2(−)* double mutants was at least partially suppressed in *pha-4(RNAi)* animals (Fig. 4F). In wild-type animals, PHA-4 is expressed at high levels in the pharynx and rectum and at low levels in the intestine (Fig. 4G,G′) (Horner et al., 1998; Kalb et al., 1998). It was previously found that ELT-2 positively regulates *pha-4*, as forced expression of *elt-2* causes widespread activation of *pha-4* (Kalb et al., 1998). Paradoxically, *pha-4* transcript levels were elevated in *elt-2(−)* single mutants and *elt-7(−);elt-2(−)* double mutants (Fig. S16). Using a CRISPR-tagged PHA-4::GFP endogenous reporter, we showed that PHA-4 was indeed upregulated in the intestine of *elt-2(−)* animals (Fig. 4H,H′), and depleting *elt-7* in *elt-2(−)* animals further enhanced this effect (Fig. 4I,I′). These findings suggest that ELT-2 serves dual roles as both an activator and a repressor of *pha-4* depending on its expression levels. Thus, it appears that upregulation of *pha-4* in the midgut in the absence of ELT-2 and ELT-7 activates sporadic ectopic pharyngeal gene activity. Supporting our model, PHA-4 target genes have been shown to be regulated in part by PHA-4 binding affinity and occupancy (Fakhouri et al., 2010; Gaudet et al., 2004). Taken together, our results show that the boundaries of regulator state domains along the digestive tract of *C. elegans* are established, at least partly, by transcriptional repression mediated by ELT-2/7 in the intestine (Fig. 4J).

### END-1 and ELT-7 establish the boundary between the valve and intestinal tubes

**Fig. 5.**
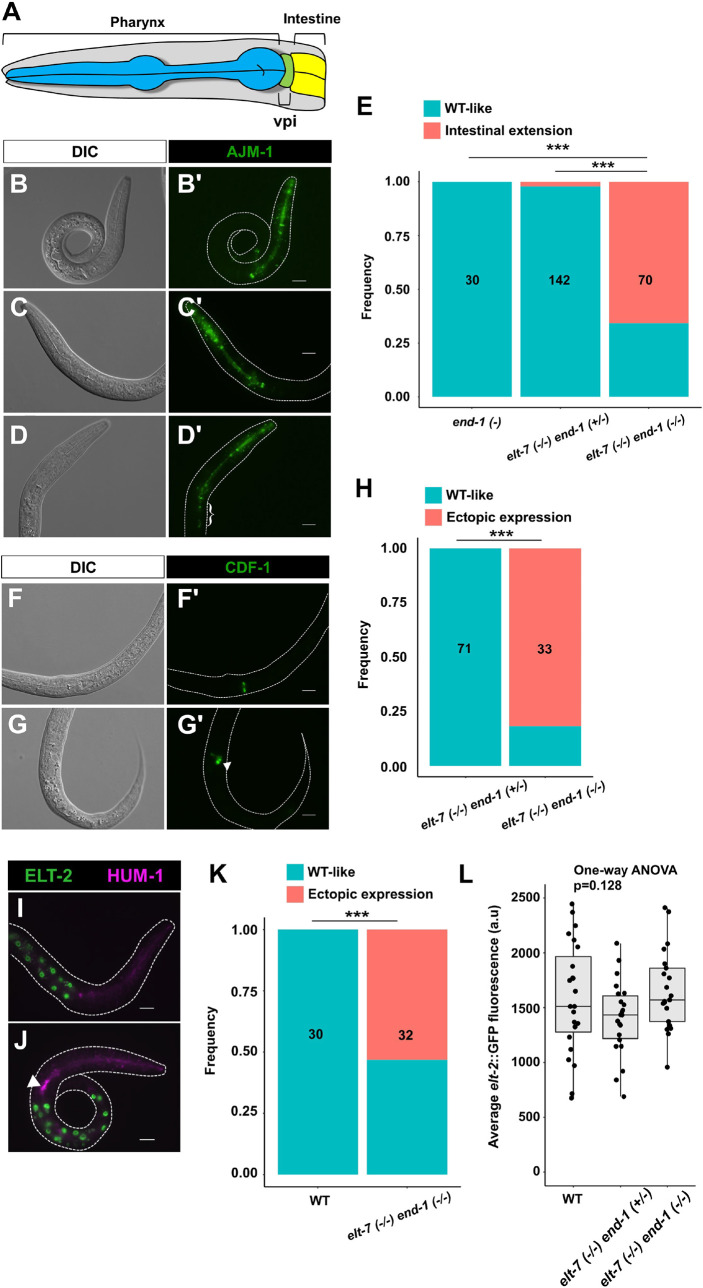
**ELT-7 and END-1 function synergistically to repress ectopic expression of valve cell markers in the anterior gut.** (A) Schematic of the anatomy of the pharynx, vpi and intestine. (B-D) The expression of the *jcIs1[ajm-1::GFP]* transgene appears similar to wild-type in *end-1(*−*)* (B,B′) and *elt-7(*−*/*−*) end-1(+/−)* (C,C′) animals. *elt-7(*−*) end-1(*−*)* (D,D′) shows caudal expansion of *jcIs1* expression into the anterior gut (curly bracket). (E) The frequency of animals exhibiting ectopic expression of *jcIs1*. (F,F′) *cdf-1::GFP* expression is restricted to the vpi in *elt-7(*−*) end-1(+/−)* animals. (G,G′) Ectopic expression of *cdf-1* reporter is observed in *elt-7(*−*/*−*) end-1(*−*/*−*)* animals (arrowhead). (H) The frequency of animals with ectopic *cdf-1::GFP* expression. (I) HUM-1 is highly expressed in the vpi in a wild-type animal as revealed by a CRISPR-tagged endogenous reporter. (J) Ectopic expression of *hum-1* is observed in *elt-7(*−*) end-1(*−*)* (arrowhead). The intestinal cells are marked by *elt-2::GFP*. (K) Frequency of animals with ectopic *hum-1::RFP* expression. (L) The expression of ELT-2 is not altered in *elt-7(*−*/*−*) end-1(+/−)* and *elt-7(*−*/*−*) end-1(*−*/*−*)*, compared with wild-type L1 larvae. a.u., arbitrary units. All scale bars: 10 μm. For panels E,H,K, the number of animals scored is indicated in each graph. ***P≤0.001 (Fisher's exact test).

The foregoing results suggest that the core regulators involved in gut differentiation (END-1, ELT-2 and ELT-7) regulate faithful differentiation of cells in the digestive tract. The pharynx and the intestine are linked by the pharyngeal-intestinal valve (vpi), which consists of six cells arranged into three rings (Rasmussen et al., 2013) (Fig. 5A). In wild-type worms, *ajm-1*::*GFP* (*jcIs1* transgene) is strongly expressed through the adherens junctions lining the lumen of the pharynx and the vpi, and the expression sharply drops to low levels starting at the anterior-most ring of the intestine, and remains low throughout the entire length of the animal (Köppen et al., 2001; Sommermann et al., 2010). However, although *end-1(−/−)* and *elt-7(−/−) end-1(*+/−*)* animals showed wild-type *ajm-1* expression pattern, the *ajm-1* signal was markedly elevated in the anterior intestinal terminus of *elt-7(−/−) end-1(−/−)* animals (Fig. 5B-E). Additionally, we observed ectopic expression of two vpi markers, *cation diffusion facilitator-1* (*cdf-1*) (Fig. 5F-H) and *heavy chain, unconventional myosin-1* (*hum-1*) (Fig. 5I-K), in the anterior terminus of *end-1(−/−) elt-7(−/−)* larvae.

It has previously been shown that worms lacking *elt-2*, similar to *elt-7(−/−) end-1(−/−)* animals, exhibit striking caudal extension of the valve cell markers (Sommermann et al., 2010). We found that ELT-2 was expressed at wild-type levels in *elt-7(−/−) end-1(−/−)* larvae, owing to its positive autoregulation (Fig. 5L). This suggests that the expansion of vpi gene expression in the intestine that we observed in *elt-7(−/−) end-1(−/−)* animals may be independent of ELT-2 function. It is currently unclear whether the expression of vpi reporters in the intestine reflects bona fide transformation of gut cells into valve-like cells or aberrant development of vpi and mispositioning of excess valve cells. Regardless, our results demonstrate the important roles of intestinal GATA factors in the development of a properly patterned digestive tract.

## DISCUSSION

The development of the *C. elegans* endoderm provides a powerful system to study the regulatory logic underlying cell specification and differentiation. In this study, we report four major findings: (1) the hierarchical organization of and feedforward regulatory relationship between GATA factors promote rapid lockdown of endodermal cell fate during *C. elegans* early embryogenesis; (2) END-1 participates in both specification and differentiation and mediates a smooth regulatory state transition; (3) ELT-2 and ELT-7 repress expression of *pha-4* in the midgut to establish the regulatory state boundary between the pharynx and the intestine; and (4) END-1, ELT-7 and ELT-2 repress the characteristics of vpi cell fate at the anterior gut terminus, further defining the spatial domains of the foregut and midgut. Our study therefore provides an important insight into the regulatory circuits that direct the specification-to-differentiation transition and subsequent restriction and maintenance of differentiation patterns during development.

### Architecture of the *C. elegans* endoderm regulatory cascade

**Fig. 6.**
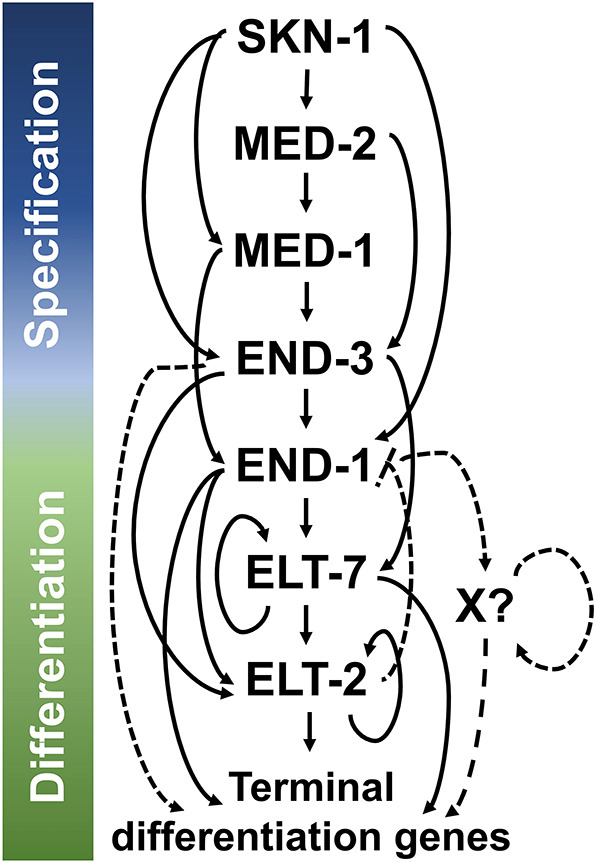
**Current model for the *C. elegans* endoderm GRN.** Solid lines indicate known interactions identified biochemically or implied genetically, whereas dashed lines represent proposed interactions. SKN-1 initiates the GATA-driven cascade. END-1 and END-3 are also regulated by non-GATA TFs, including SPTF-3 (Sullivan-Brown et al., 2016), PAL-1 (Maduro et al., 2005b), PLP-1 (Witze et al., 2009) and POP-1 (Maduro et al., 2005b), which are omitted from this model for simplicity. See text for details.

This study provides a comprehensive overview of the core endoderm regulatory cascade, with six GATA factors acting through reiterated, sequential feedforward loops (Fig. 6). At the top of the cascade, maternally provided SKN-1 turns on the *med* and the *end* genes (Maduro et al., 2001, 2005b). Although the MED-1 and MED-2 protein sequences are nearly identical, we found distinguishable contributions between the two paralogs, with MED-2 acting upstream of MED-1. Indeed, embryos lacking MED-1 show a weaker loss-of-gut phenotype than those lacking MED-2 when SKN-1 function is impaired (Maduro et al., 2007). Moreover, *med-2* is expressed slightly earlier than *med-1* (Maduro et al., 2007; Tintori et al., 2016), suggesting that the two genes are differentially regulated, as we have observed in this study.

In the E blastomere, SKN-1 and the MEDs collaboratively activate END-3. MED-1/2 and END-3 in turn activate END-1, the next step in the cascade. Although the two endoderm-specifying factors, END-1 and END-3, perform largely overlapping functions, our data suggest that END-3 alone is sufficient to direct endoderm specification and suppress improper mesectodermal development, consistent with the lack of a detectable phenotype in *end-1(−)* single mutants. Interestingly, we found that END-1, which is poised at the interface between specification and differentiation, works synergistically with ELT-2 and ELT-7 to promote endoderm differentiation (Fig. 6). Supporting our model, *in vitro* gel-shift assays demonstrate the binding of END-1, ELT-7 and ELT-2 to TGATAA sites, which are highly enriched in the promotors of intestinal genes (Du et al., 2016; McGhee et al., 2009; Wiesenfahrt et al., 2016). Remarkably, END-1 is able to initiate endoderm differentiation in *Xenopus* embryos, demonstrating its role as a potent organ selector (Shoichet et al., 2000). Hence, it appears that specification and differentiation involve a bona fide handoff of regulatory events by END-1.

Our results indicate that multiple factors (END-1, ELT-7 and ELT-2) act synergistically to promote endoderm morphological differentiation, challenging the notion that ELT-2 is the dominant ‘organ identity factor’ for intestinal differentiation (Fukushige et al., 1998; McGhee, 2007; McGhee et al., 2007, 2009). *elt-2(−)* animals produce an apparently complete, though defective, organ and express most gut-specific genes. The essential function for ELT-2 in widespread gut morphological differentiation is revealed only when its strongly synergistic action with ELT-7 function is eliminated (our observations) (Sommermann et al., 2010). Although the majority of hatched *end-1(−);elt-2(−)* larvae contain a continuous lumen, many *elt-7(−) end-3(−)* animals show significant gut defects, demonstrating that ELT-2 alone is insufficient to drive robust gut development. ELT-7, when expressed under the control of the *end-1* and *elt-2* promotors, can replace all other GATA factors in the GRN (Dineen et al., 2018). Additionally, overexpression of ELT-7 may cause widespread transdifferentiation of fully differentiated post-mitotic cells, showing that ELT-7 is a potent driver of intestinal differentiation (Riddle et al., 2013, 2016).

### Regulatory logic of a developmental GRN

Our results support the conclusion that the *C. elegans* endoderm GRN comprises a recursive series of feedforward steps, culminating in rapid terminal differentiation (Fig. 6). Each GATA factor in the cascade receives redundant activating inputs acting through an ‘OR’ logic gate. Consequently, any single mutation in the regulatory cascade is largely phenotypically silent, with the exception of *elt-2(−)*; however, even *elt-2(−)* animals contain what appears to be a well-differentiated, albeit dysfunctional, intestine. Coherent feedforward motifs of the type that we observed reiteratively in the endoderm GRN are ubiquitous in developmental GRNs. Such a network configuration appears to result in a rapid response to an activating signal and a delayed response when the inputs are removed (termed sign-sensitive delay), thereby prolonging the effect of a transient activator (Mangan and Alon, 2003). Additionally, such feedforward loops are effective at buffering the system against stochastic noise, thereby ensuring developmental robustness (Chepyala et al., 2016; Gui et al., 2016; Maduro, 2015). This design principle appears to be crucial to ensure timely and robust activation of *elt-7* and *elt-2*, as we and others have shown (Boeck et al., 2011; Dineen et al., 2018; Maduro et al., 2015). Timely onset of *elt-2* appears to be crucial, as its delay in early embryos has been shown to cause sustained metabolic defects in larvae, despite later reattainment of wild-type ELT-2 levels (Maduro et al., 2015; Choi et al., 2017).

Our results suggest that END-1 (and potentially END-3) mediates an all-or-none switch in the differentiation program in the absence of ELT-2 and ELT-7. END-1 straddles both specification and differentiation, being buttressed by END-3 upstream and ELT-2/7 downstream. The only difference in these END-1 functions appears to be its timing of action and partnership with another regulatory factor. Regulatory nodes in early specification can indeed directly control morphogenetic events in various contexts (Davidson, 2010; Zhu and Rosenfeld, 2004). Interestingly, although END-1 expression declines shortly after gastrulation, we observed a persistent reduction in *act-5* expression in postembryonic larvae lacking *end-1*. One possible explanation for this observation, and the finding that patches of well-differentiated gut arise in *elt-7(−);elt-2(−)* double mutants long after END-1 becomes undetectable, might be that END-1 targets an unidentified factor (indicated by X in Fig. 6) that directs differentiation in at least a subset of endodermal progenitors. Like ELT-2/7, expression of the putative factor X would be expected to be maintained through a positive autoregulatory loop, thereby continuing to promote gut differentiation after the expression of END-1 has subsided (Fig. 6) (Sommermann et al., 2010). Alternatively, END-1 may directly activate differentiation gene batteries in early embryos, and that regulatory state might be sustained through the propagation of epigenetic memory. This priming mechanism has recently been demonstrated in the specification of the ASE sensory neurons in *C. elegans* (Charest et al., 2020). Two transiently expressed T-box factors, TBX-37 and TBX-38, lock their target, *lsy-6*, in a transcriptionally active state during early embryogenesis, priming it for activation in restricted neuronal lineage (Charest et al., 2020). In mammals, Pax-7 initiates myogenic specification and differentiation. It is of relevance to note that many enhancers of the target genes of Pax-7 retain epigenetic signatures and remain active even in the absence of the initial activator (Zhang et al., 2020).

Our preliminary results showed that knocking down *elt-2* causes a slight but significant increase in *end-1* expression in early embryos; however, we did not observe obvious perdurance of END-1 when ELT-2 and ELT-7 were depleted, although we cannot rule out the possibility that the modest negative feedback we observed resulted from incomplete RNAi penetrance. Nevertheless, we propose that this modest feedback inhibition may function to facilitate the transition of regulatory states and ensure that the developmental process moves inexorably forward. For example, repression of an early cardiac specification factor, Bmp2, by the Nkx2-5 homeodomain factor is necessary for proper morphological development of the heart in mice (Prall et al., 2007). Such transcriptional repression is also frequently used to install spatial subdivision of regulatory states (Davidson, 2010). As we have shown above, ELT-2, ELT-7 and END-1 may repress alternate cell fates in the midgut and define the boundaries of the digestive tract. It is noteworthy that structurally similar regulatory circuits are repeatedly deployed in different biological networks while performing similar functions. Thus, the functional output of a GRN depends not only on the specificity of the TFs, but also the underlying circuit architecture (Davidson, 2010; Peter, 2020).

### Rapid rewiring of the endoderm GRN in *Caenorhabditis*

How might a regulatory system of the type described here evolve? Effectors acting on terminal differentiation gene batteries, such as ELT-2 and PHA-4, are widely conserved across the animal kingdom, whereas the upstream inputs into GRNs appear to be recent innovations that arose during the radiation of the Elegans supergroup within the *Caenorhabditis* genus (Maduro, 2020). The *end* and *med* genes have been proposed to have arisen from the duplication of *elt-2*. Hence, gene duplication, coupled with *cis*-regulatory changes, may have led to the emergence of new circuitry and rewiring of the endoderm GRN in nematodes.

Intercalation of the MEDs and ENDs in the cascade may serve to buffer the system against environmental variation and developmental noise by freeing ELT-2 from direct control of SKN-1, which has been shown to play conserved pleiotropic roles in stress response and lifespan regulation (reviewed by Ewe et al., 2020, 2021). As we and others have shown, robust induction of *elt-2* is crucial to ensure the viability and fitness of the animals (Maduro et al., 2015; Raj et al., 2010). Moreover, the deployment of MEDs and ENDs in the sequential hierarchy may allow canalization of the endoderm lineage by rapidly establishing its regulatory state in the E blastomere (Peter and Davidson, 2011). Consequently, this structure may enable increased developmental speed and early specification of the founder cells in *Caenorhabditis* species.

## MATERIALS AND METHODS

### *C. elegans* cultivation and genetics

Worm strains were cultured using standard procedures (Brenner, 1974) and all experiments were performed at room temperature (20-23°C). All genetic manipulations were performed according to standard techniques (Fay, 2013). *him-5(−)* or *him-8(−)* mutations were introduced into some strains to generate males used in crosses. Mutations and transgenes were validated by PCR and sequencing. Some lethal mutations were balanced using structurally defined balancers with fluorescent and phenotypic markers (Dejima et al., 2018). *elt-2(ca15)* was balanced with an extrachromosomal transgenic array, *irEx404 [unc-119::CFP, elt-2(+)]*. Animals that had lost the *elt-2(+)* rescuing array were CFP-negative and underwent developmental arrest (Sommermann et al., 2010). The mutations in the GATA genes are described in Table S1 (see also www.wormbase.org) and a complete list of strains used in this study is provided in Table S3. A summary of PCR primers used to detect mutations is given in Table S2.

### Immunofluorescence analysis

Antibody staining with methanol-acetone fixation was performed as previously described (Sommermann et al., 2010). The antibodies MH27 (1:1000, AB_531819, Developmental Studies Hybridoma Bank), MH33 (1:1000, AB_528311, Developmental Studies Hybridoma Bank) and 455-2A4 (1:1000, AB_2618114 , Developmental Studies Hybridoma Bank) were used to detect AJM-1, IFB-2 and ELT-2, respectively. Alexa Fluor 488 goat anti-mouse secondary antibody (Abcam; ab150113) was used at 1:1000 dilution.

### RNAi

RNAi feeding clones were obtained from the Ahringer (Kamath et al., 2003) or the Vidal (Rual et al., 2004) libraries. The bacterial strains were inoculated overnight at 37°C in LB media containing 50 μg/ml ampicillin. The culture was then diluted 1:10 and incubated for an additional 4 h. Next, 1 mM of isopropyl β-D-1-thiogalactopyranoside (IPTG) was added to the bacterial culture and 100 μl was seeded into 35 mm nematode growth medium (NGM) agar plates containing 1 mM IPTG and 25 μg/ml carbenicillin. For simultaneous knockdown of *elt-2* and *elt-7*, the two bacterial strains, each expressing dsRNA for one gene, were concentrated and resuspended in 1 ml of LB at a 1:1 ratio before seeding the NGM plates. Seeded plates were allowed to dry for 48 h before use. Next, 10-20 L4 animals were placed on the RNAi plates. After 24 h, the animals were transferred to fresh RNAi plates and allowed to lay eggs. The progeny were then collected for analyses.

### Imaging and fluorescence quantification

The animals were immobilized using 10 mM levamisole and mounted on 4% agarose pads. Images were acquired, typically at 60×, using a Nikon Eclipse Ti-E inverted microscope fitted with an ORCA-Flash2.8 camera. For expression studies, a maximum intensity *z*-projection was generated with the Nikon NIS-Elements AR v4.13.05. Images were then analyzed using ImageJ or Imaris v9.7.2.

### Reverse transcription quantitative PCR (RT-qPCR)

RNA was extracted from synchronized L1 animals using Monarch Total RNA Miniprep Kit (TS010S, New England Biolabs). cDNA synthesis was performed using SuperScript III First-Strand Synthesis SuperMix (Thermo Fisher Scientific, 18080400). qPCR was performed using BioRad CFX96 Real-Time System. Each 15 µl reaction contained cDNA, primers and PowerUp SYBR Green Master Mix (Thermo Fisher Scientific, A25743). The data were analyzed using the standard 2^−ΔΔCT^ method (Livak and Schmittgen, 2001). The primer sequences used were: *act-1*, 5′-TCCATTGTCGGAAGACCACG-3′ and 5′-GGTGACGATACCGTGCTCAA-3′; *act-5*, 5′-GTCACTCACACCGTTCCAATC-3′ and 5′-GTGAGGATCTTCATCATGTAGTCG-3′.

### Modeling endoderm gene regulatory circuits

The topology of the gene circuits with temporal information was written as a system of differential equations, with expression of each factor dependent on the concentration of its activators (see Supplementary Materials and Methods and Table S4). The gene cascade is initiated by SKN-1, which was modeled as a square wave in the EMS blastomere. Similarly, the positive inputs of (phosphorylated) POP-1 into *end-3* and *end-1* were modeled as a square wave in the E blastomere (23 mins after the four-cell stage). Model runs were calculated as time-discretized Euler approximations (Hahn, 1991) with time steps of 0.01 s. An iterative least-squares algorithm following a modified Gauss–Newton method (Ruhe, 1979; Yip et al., 2010) was used to fit the model parameters to published transcriptomics data (Baugh et al., 2003; Tintori et al., 2016). The performance of the final model was then evaluated by comparing the predicted phenotypes of the single mutants with published results (Boeck et al., 2011; Dineen et al., 2018; Maduro et al., 2005a, 2015). Finally, predictions of *elt-2* activation (a readout for the commitment to E fate) in the mutant combinations were generated by holding the concentration of knockout genes at zero and otherwise running the model as described. The source code for this analysis is available at https://github.com/RothmanLabCode/endoderm_GRN_model.

### Statistics and figure preparation

Statistical analyses were performed using R software v3.4.1 (https://www.r-project.org/). The specific statistical tests are reported in the figure legends. Plots were generated using R package ggplot2 or Microsoft Excel. Figures were assembled in Inkscape v0.92.4 (https://inkscape.org/).

## Supplementary Material

10.1242/develop.200337_sup1 Supplementary information
