# Supplementary material for: Feedforward regulatory logic controls the specification-to-differentiation transition and terminal cell fate during Caenorhabditis elegans endoderm development
Source: Development. 2022 Jun 27;149(12):dev200337. doi: 10.1242/dev.200337 (PMC10656426; doi:10.1242/dev.200337)
Supplement: Supplementary information [file develop-149-200337-s1.pdf]

## Supplementary Materials and Methods

Description of computational model

The interactions between the GATA factors (Figure 6), as well as POP-1 activation of end-3 and end-1, were written as a system of differential equations. Each factor is expressed according to the concentration of its activators multiplied by respective coefficients $a$, representing the strength of activation (see Supplementary File 2). The activation is not instant, but delayed by a time interval $\tau$, which accounts for the time required for transcription, translation, and re-localization to the nucleus. SKN-1 and POP-1 activation occur as square waves in EMS and E blastomere, respectively. ELT-7 and ELT-2 self-activate via feedback loops of strength $f_1$ and $f_2$, respectively, after surpassing arbitrary concentration thresholds $\Phi_1$ and $\Phi_2$, respectively. Finally, all factors are degraded at the same rate $\delta$. The resulting system of equations is as follows.

$$(1)\ \frac{d[MED2]}{dt} = a_1[SKN1]_t - \delta[MED2]_t$$

$$(2)\ \frac{d[MED1]}{dt} = a_2[SKN1]_t + a_5[MED2]_{t-\tau} - \delta[MED1]_t$$

$$(3)\ \frac{d[END3]}{dt} = a_3[SKN1]_t + a_9[POP1]_t + a_6[MED2]_{t-\tau} + a_7[MED1]_{t-\tau} - \delta[END3]_t$$

$$(4)\ \frac{d[END1]}{dt} = a_4[SKN1]_t + a_{10}[POP1]_t + a_8[MED1]_{t-\tau} + a_{11}[END3]_{t-\tau} - \delta[END1]_t$$

$$(5)\ \frac{d[ELT7]}{dt} = a_{12}[END3]_{t-\tau} + a_{13}[END1]_{t-\tau} + f_1([ELT7]_{t-\tau} > \Phi_1)[ELT7]_{t-\tau} - \delta[ELT7]_t$$

$$(6)\ \frac{d[ELT2]}{dt} = a_{14}[END1]_{t-\tau} + a_{15}[ELT7]_{t-\tau} + f_2([ELT2]_{t-\tau} > \Phi_2)[ELT2]_{t-\tau} - \delta[ELT2]_t$$

Model parameters (Supplementary File 2) were determined by fitting to transcriptomics data by a custom algorithm written in R following an iterative least-squares method. Parameter guesses were provided, and the model was run using an Euler approximation with 0.01 s time steps. The model fit was measured as the sum of squared differences between model predictions and transcriptomics data. Randomly selected parameter values were randomly changed, and the

Development • Supplementary information

model was run again. If the resulting sum of squares was lower, the new parameter values were kept. This process was iterated ~$10^6$ times. Algorithm-selected parameter values were validated by checking model predictions against the published phenotypes of the single mutants (Boeck et al., 2011; Dineen et al., 2018; Maduro et al., 2015). Two parameters ($a_8$ and $a_{14}$) were adjusted manually such that the predicted phenotype of end-3(-) matched that reported in published studies (Boeck et al., 2011; Maduro et al., 2005a). The source code is available on https://github.com/RothmanLabCode/endoderm_GRN_model.

Development • Supplementary information

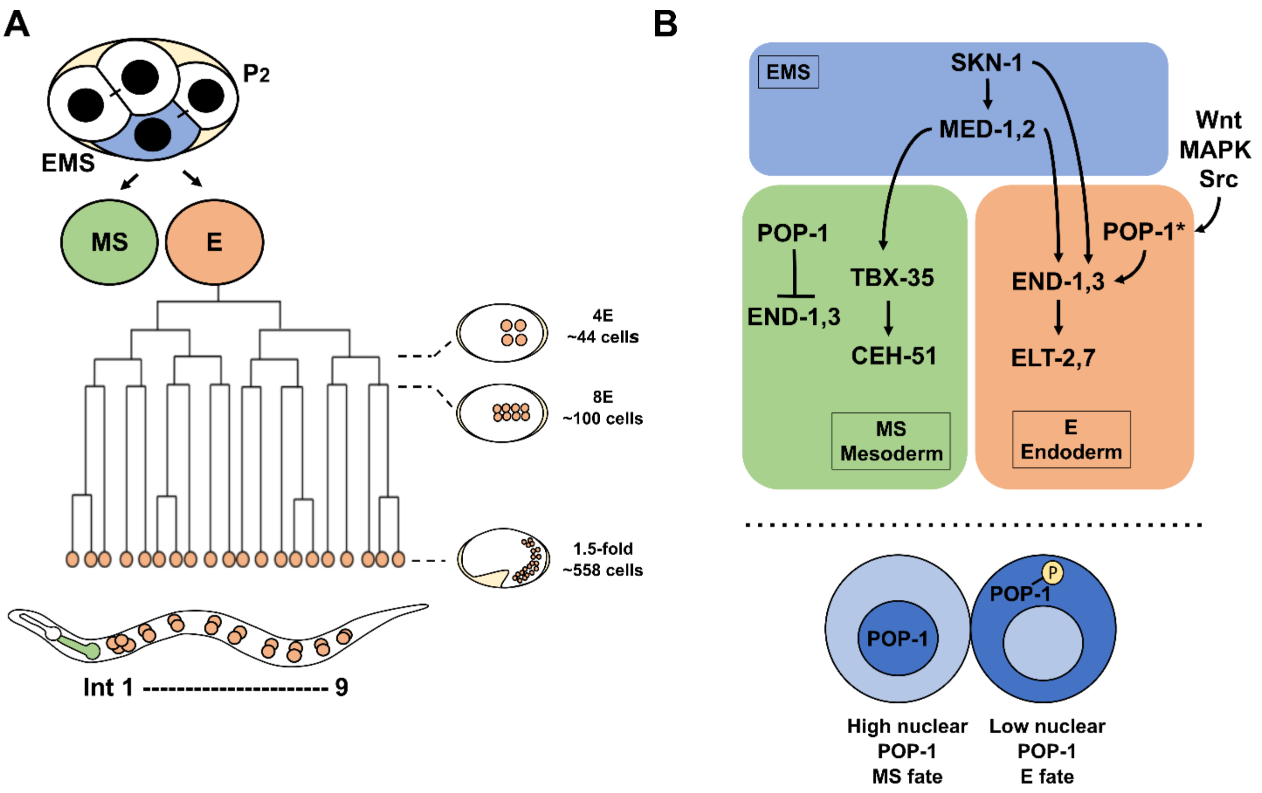


**Fig. S1.** ***C. elegans*** **mesendoderm development.**

(A) At the four-cell stage, EMS (blue) undergoes asymmetrical division to produce anterior MS (green) and posterior E (orange) blastomeres. Newly hatched L1s contain 20 intestinal cells arranged in nine rings (Ints). Int1 contains four cells, while the remaining eight rings contain two cells each. The MS cell gives rise mesodermal cell types, including the posterior pharynx. (B) Simplified mesendoderm GRN showing three sequential tiers of paired redundant GATA transcription factors (2 redundant MEDs → 2 redundant ENDs → 2 redundant ELTs). Maternally provided SKN-1 activates med-1 and -2, which have both a maternal and zygotic component. In MS, POP-1 represses the expression of end-1 and -3 genes. MED-1 and -2 then directly activate the expression of mesoderm-specifying factor, TBX-35. In E, however, Wnt, MAPK and Src signaling from neighboring P2 cell ultimately leads to the phosphorylation of POP-1 (indicated by *), altering its nucleocytoplasmic localization (high nuclear level in MS and low nuclear level in E; bottom panel) and converting it from a repressor to an activator of gut fate (Shetty et al., 2005). Hence, MED-1/2 and POP-1* provide redundant inputs to turn on end-1/3. END-1 and -3 subsequently activate the expression of ELT-7 and -2, both of which promote gut morphological differentiation.

Development • Supplementary information

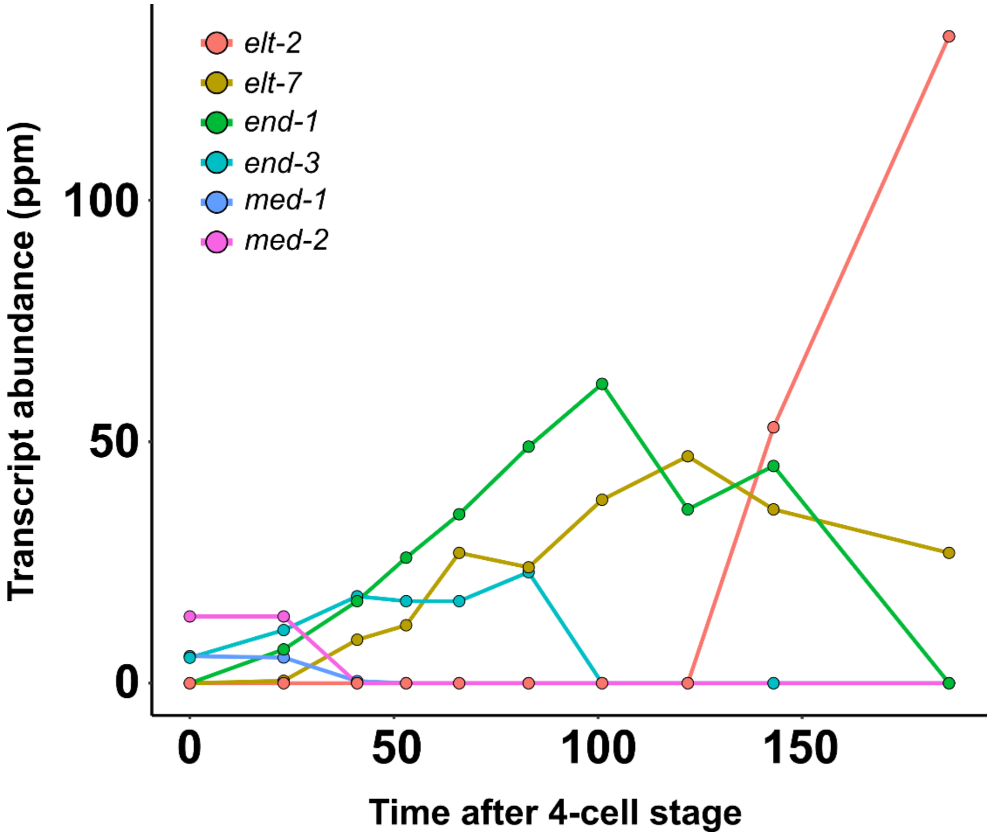


**Fig. S2. The endoderm GATA factors are deployed in temporal order.** Temporal expression of med-2, med-1, end-3, end-1, elt-7, and elt-2 revealed by microarray (Baugh et al., 2003) and single-cell transcriptomic analysis (http://tintori.bio.unc.edu/) (Tintori et al., 2016).

Development • Supplementary information

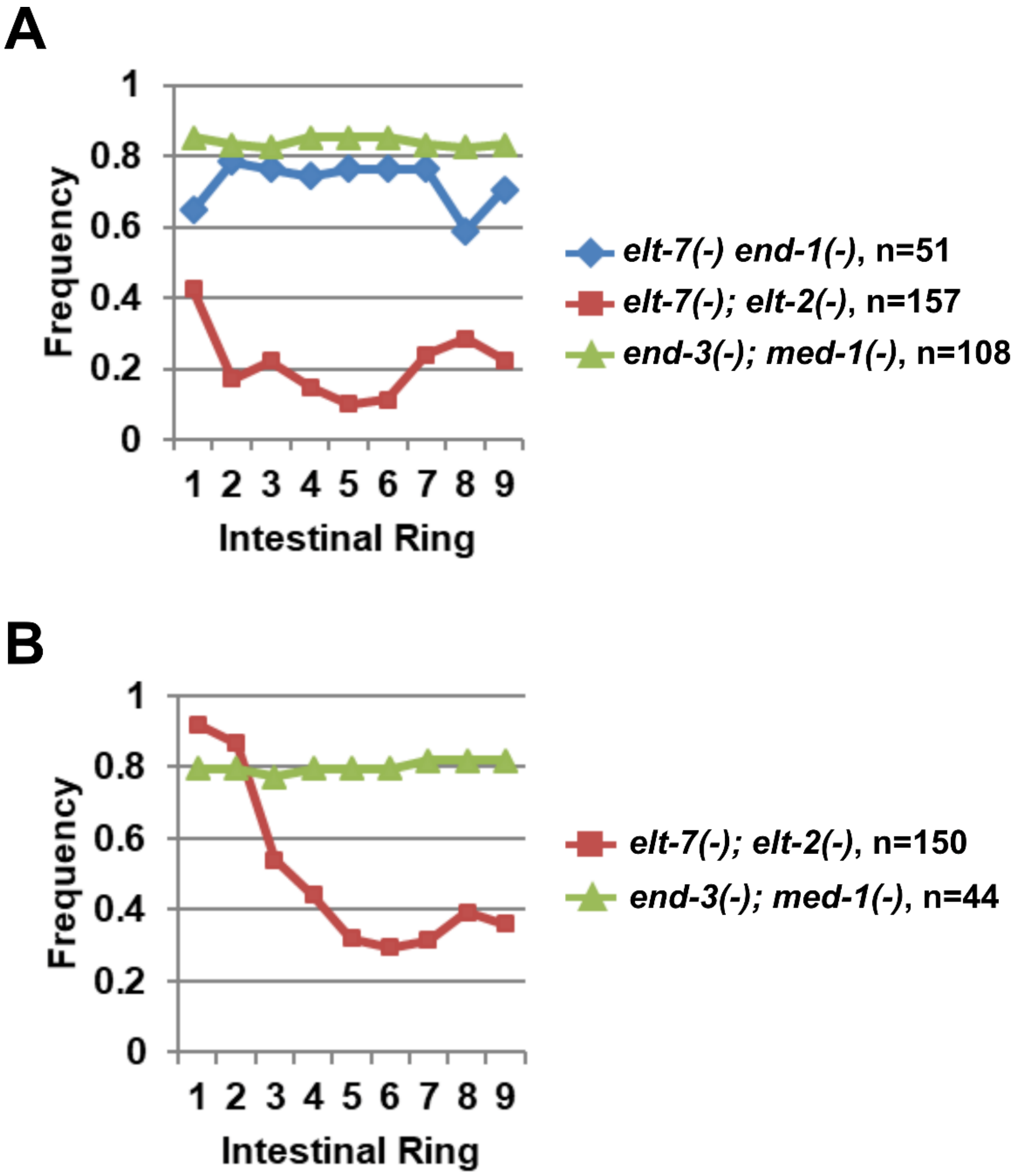


**Fig. S3. Severe gut defects in mutants lacking sequential GATA pairs.** Eliminating sequential GATA pairs causes impaired gut differentiation and aberrant expression of immunoreactive (A) IFB-2 and (B) AJM-1.

Development • Supplementary information

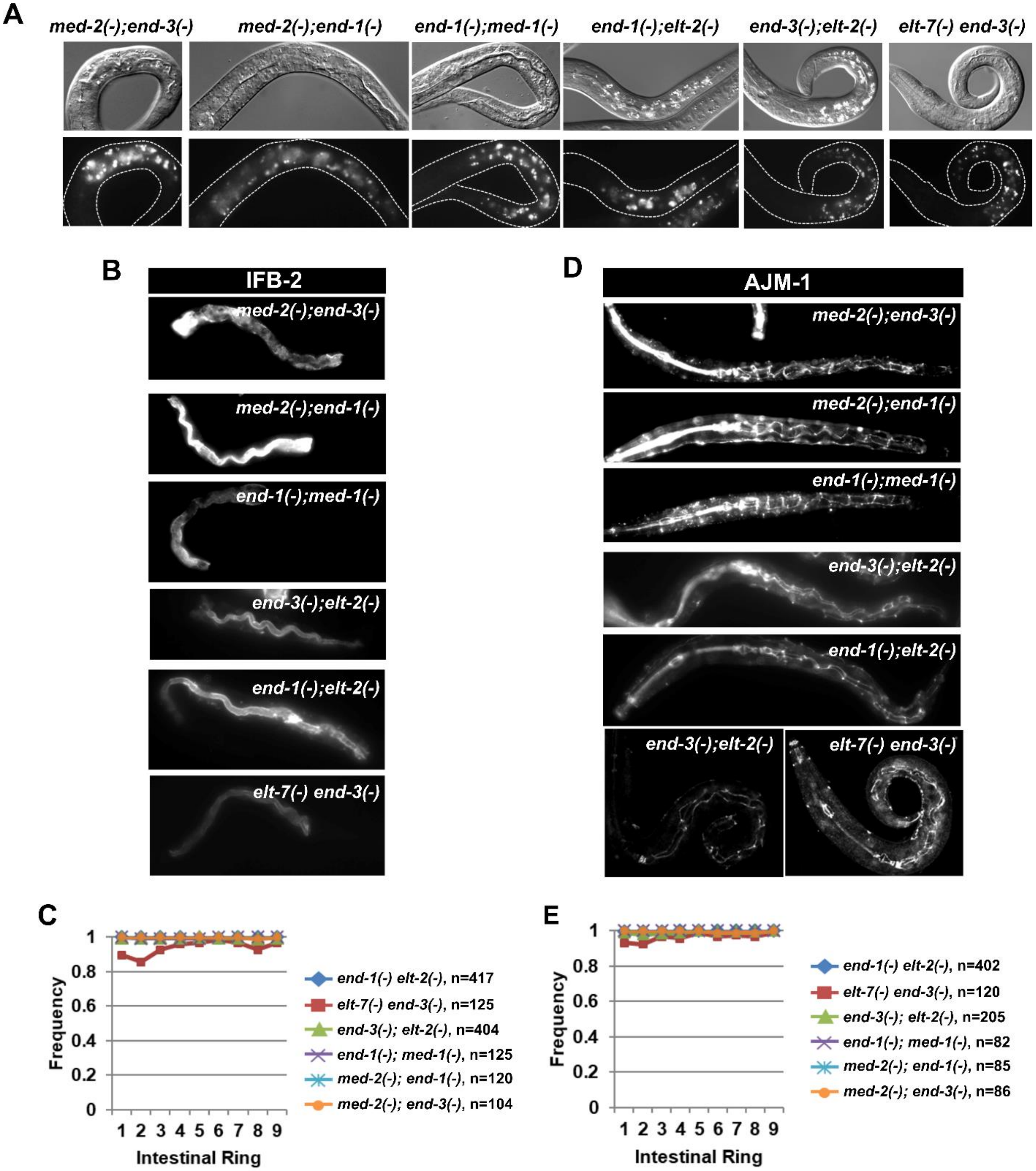


**Fig. S4. Mutants lacking alternate GATA pairs do not show apparent gut defects.**

(A) Mutants lacking alternate GATA pairs contain fully differentiated lumen (top row) and gut granules (bottom row) along the length of the animals. The same set of double mutants show wildtype expression of (B, C) immunoreactive IFB-2 and (D, E) AJM-1.

Development • Supplementary information

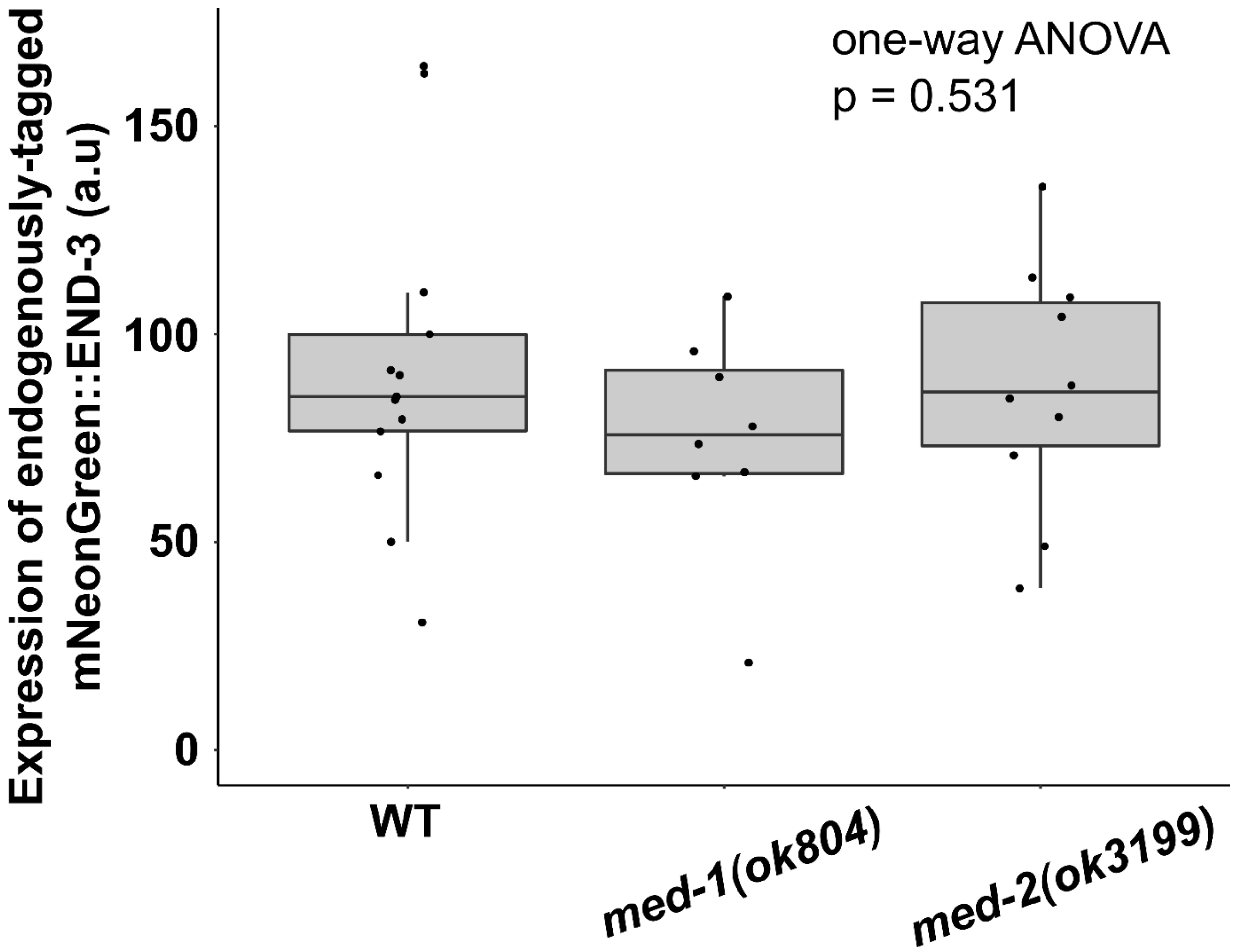


**Fig. S5. The expression of endogenously tagged END-3 reporter in the *med* mutants.** Removing med-1 or med-2 alone does not affect end-3 expression at the 4E embryonic stage.

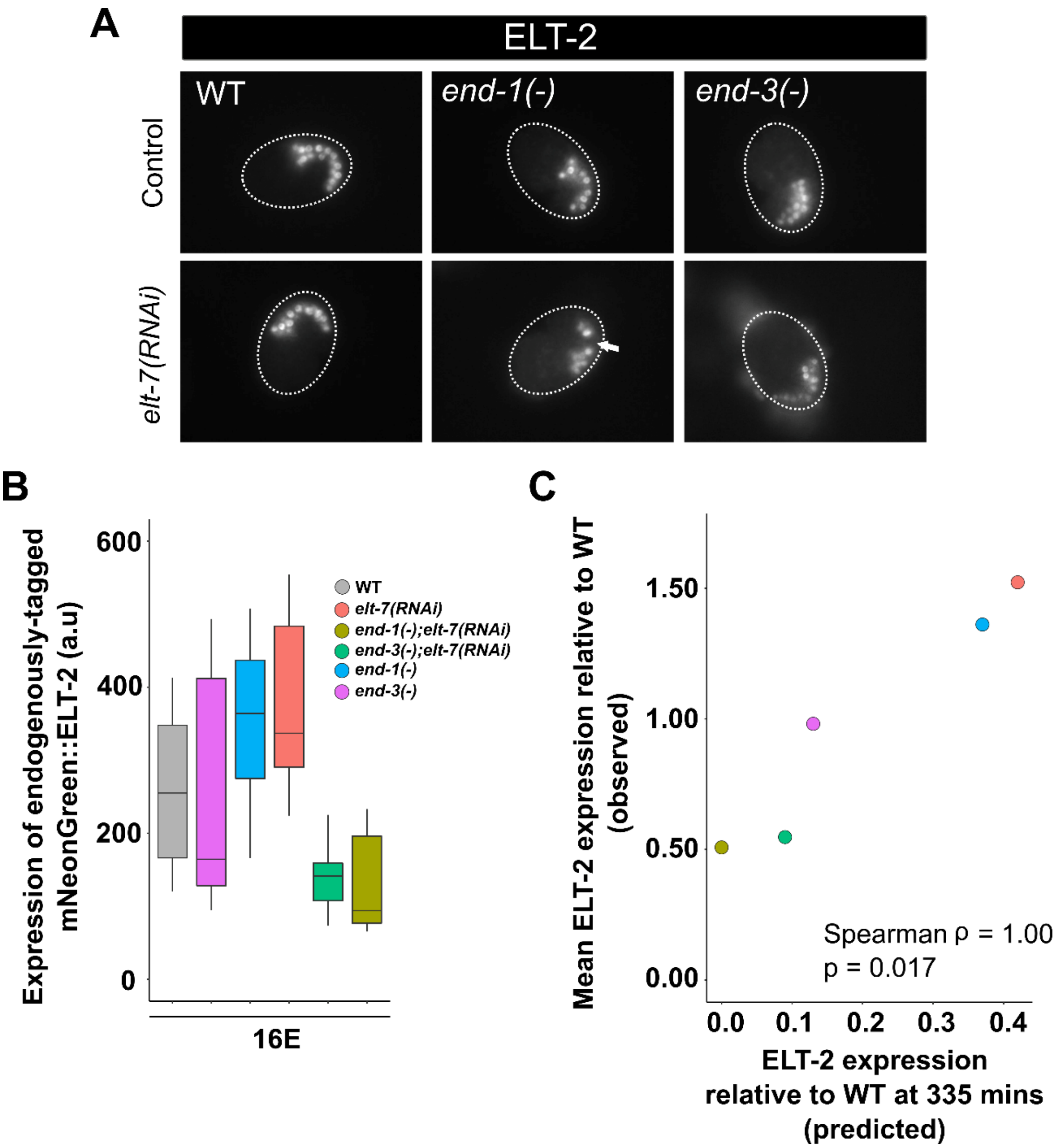


**Fig. S6. The Expression of endogenously tagged ELT-2 reporter in the GATA mutant combinations.**

(A) The expression of endogenously-tagged mNeonGreen::ELT-2 reporter in wildtype, end-3(-), end-1(-), elt-7(RNAi), end-3(-); elt-7(RNAi), and end-1(-); elt-7(RNAi) embryos at the comma stage. Note that end-1(-); elt-7(RNAi) contains a truncated gut with missing gut cells (highlighted by white arrow). (B) The quantification of ELT-2 expression in the mutant combinations at 16E embryonic stage. (C) The measured ELT-2 expression levels are strongly correlated with the predicted values deduced from the computational model of the feedforward circuits (see Figure 1K, K').

Development • Supplementary information

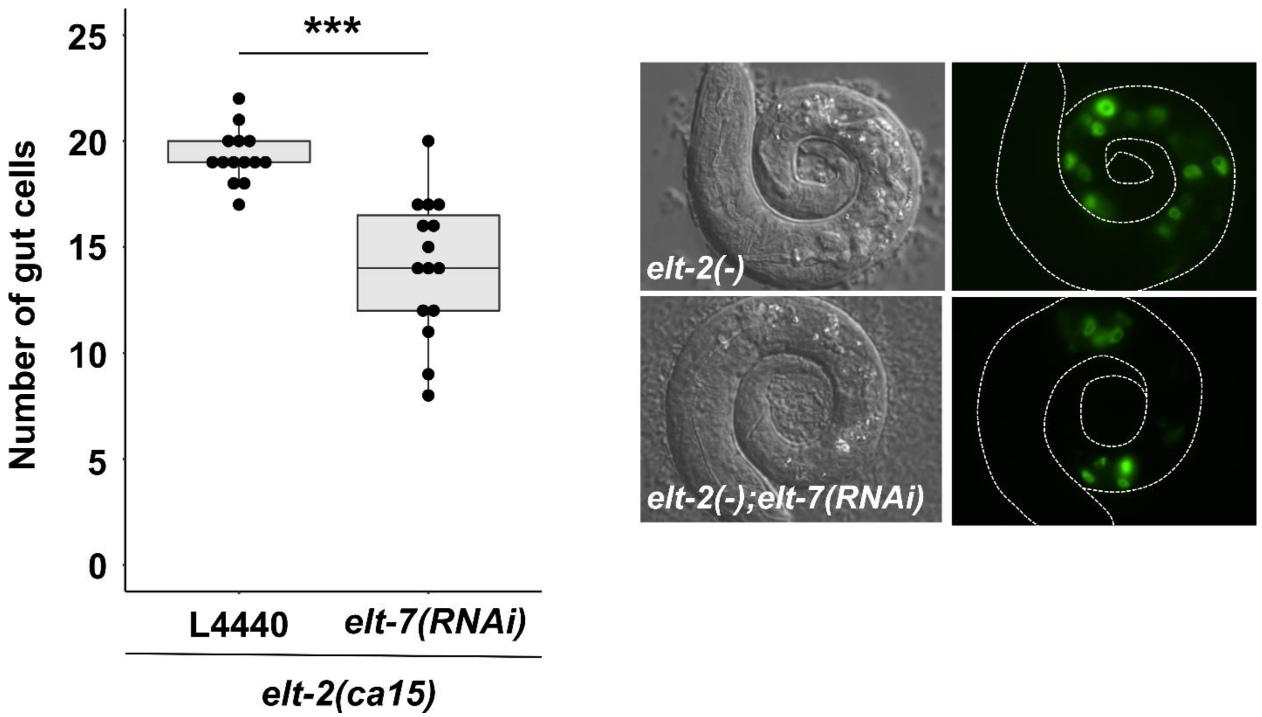


**Fig. S7. Reduced number of differentiated intestinal cells *in elt-2(-); elt- 7(RNAi)* animals.** On average, elt-2(-) animals contain 19.3 cells, while elt-2(-); elt-7(RNAi) animals contain 14.1 cells. The number of gut cells were scored by the expression of elt-2p::GFP transcriptional reporter wIs84. Note that elt-7(-); elt-2(-) chromosomal double mutant and elt-2(-); elt-7(RNAi) animals are indistinguishable in appearance with extensive gut defects, demonstrating strong penetrance of elt-7 RNAi. *** $p \leq 0.001$ by Wilcoxon tests.

Development • Supplementary information

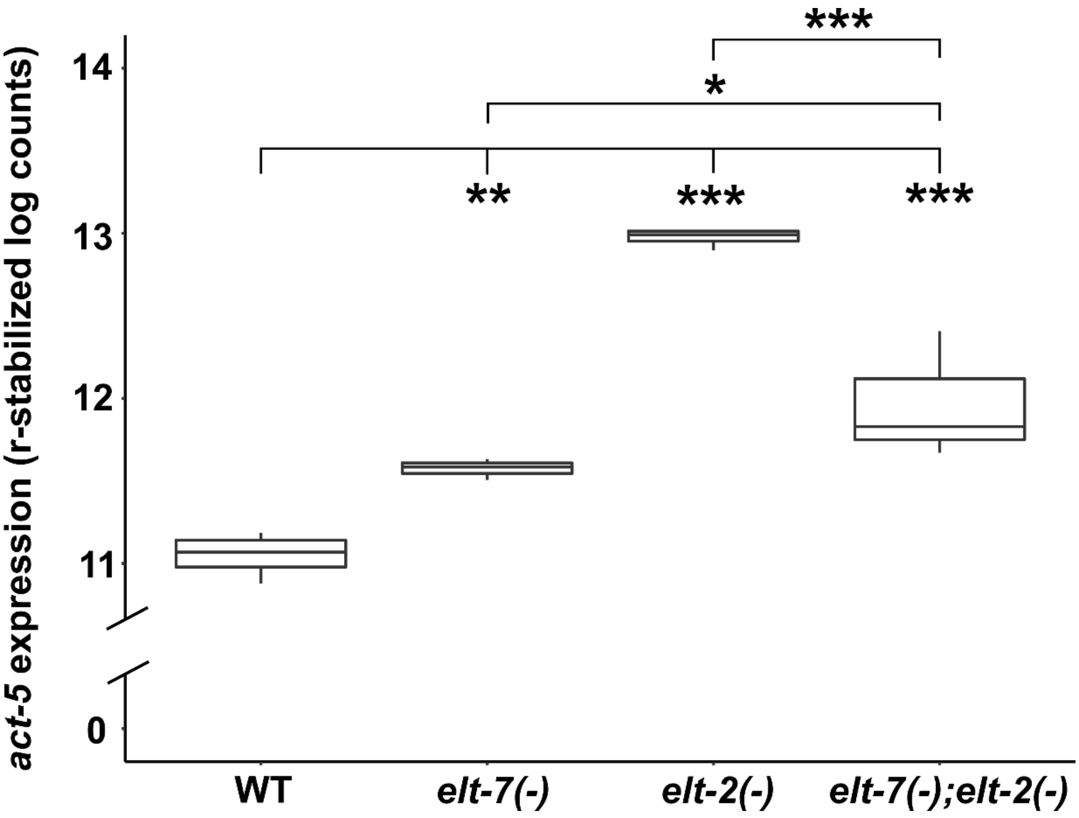


**Fig. S8. *act-5* transcript levels are upregulated in *elt-7(-)*, *elt-2(-)*, and *elt- 7(-); elt-2(-)*.** Eliminating elt-7 or elt-2 causes upregulation of act-5. The expression of act-5 is modestly reduced in elt-7(-); elt-2(-) compared to elt-2(-), suggesting that ELT-7 may also contribute to the transcriptional activation of act-5 in the absence of ELT-2. * $p \leq 0.05$, ** $p \leq 0.01$, *** $p \leq 0.001$ by parametric one-way ANOVA followed by pairwise t-tests with Benjamini & Hochberg correction. RNA-seq data was retrieved from (Dineen et al., 2018)

Development • Supplementary information

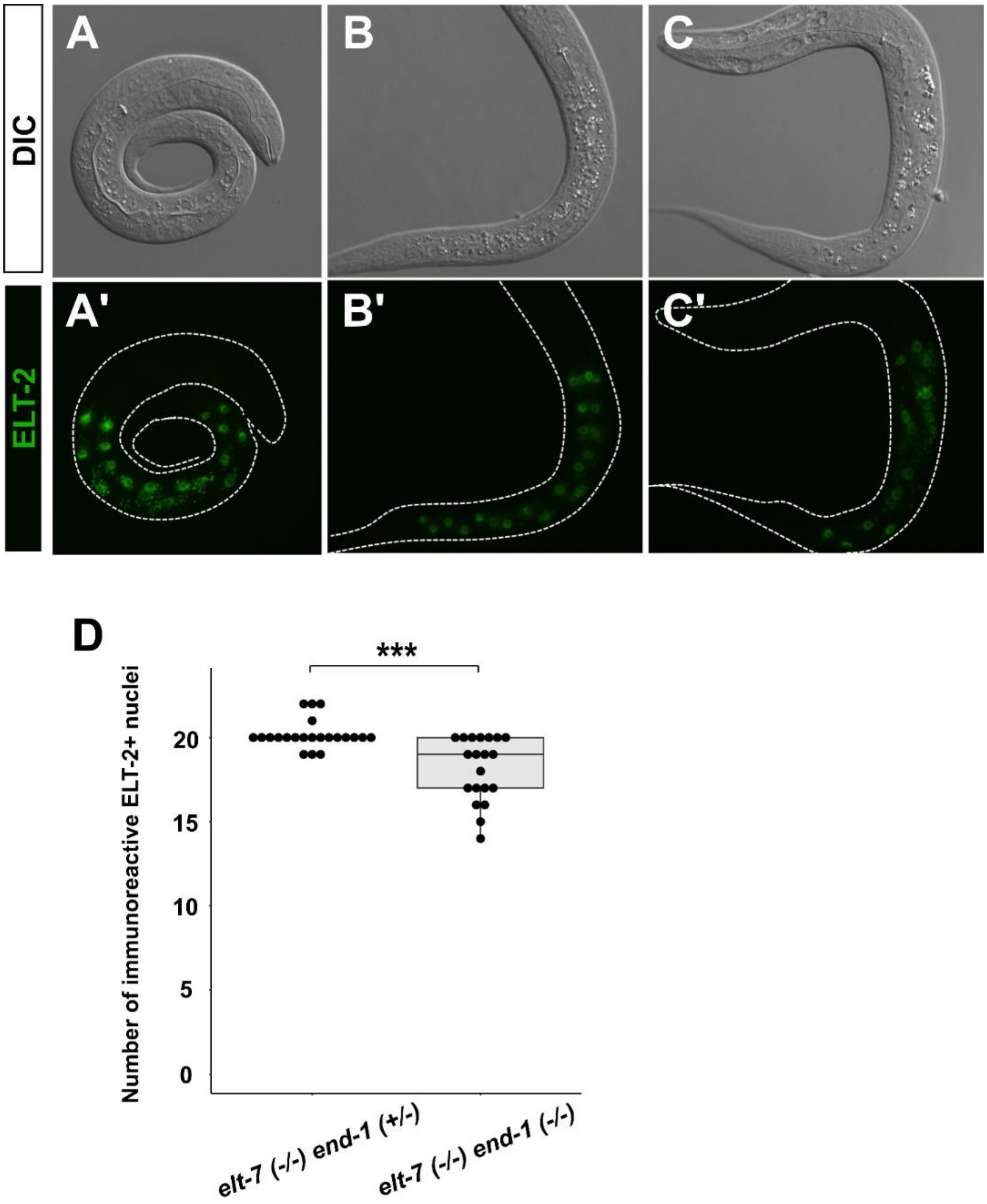


**Fig. S9. Reduced number of differentiated intestinal cells in *elt-7(-) end-1(-)* animals.** (A-C) The expression of elt-2::GFP translational reporter in (A, A') wildtype, (B, B') elt-7(-/-) end-1(+/-), and (C, C') elt-7(-/-) end-1(-/-) L1 larvae. (D) elt-7(-/-) end-1(-/-) double mutants contain fewer cells expressing immunoreactive ELT-2 than elt-7(-/-) end-1(+/-) animals.

*** $p \leq 0.001$ by Wilcoxon tests.

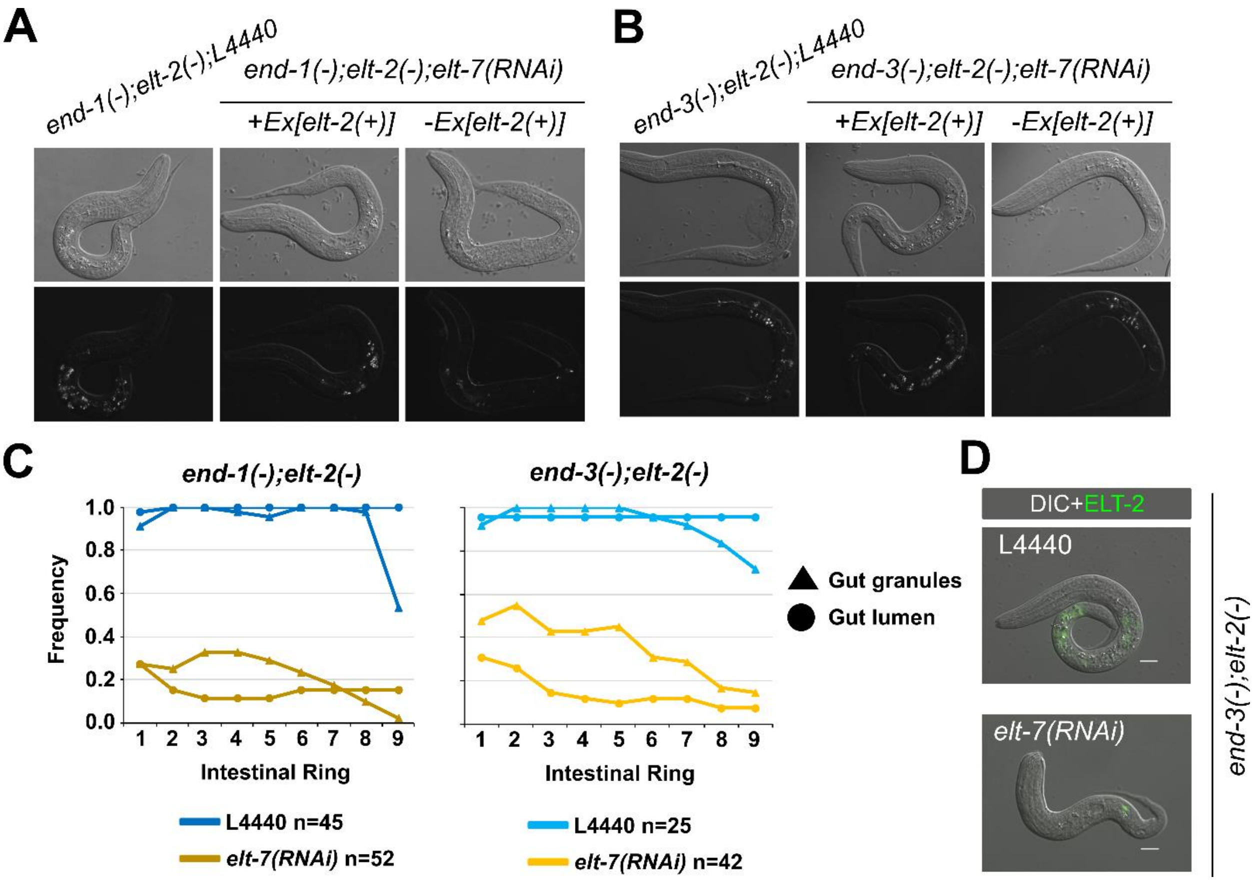


**Fig. S10. Severe gut differentiation defects in animals lacking *end-3*, *elt-7*, and *elt-2*.** (A-C) Knocking out elt-7 causes severe gut differentiation defects in both (A, C - left) end-1(-); elt-2(-) and (B, C - right) end-3(-); elt-2(-) animals. The elt-7 RNAi treated animals contain little to no visible lumen and birefringent gut granules. (D) Knocking down elt-7 in end-3(-); elt-2(-) leads to a marked decrease in the expression of elt-2 transcriptional reporter (wIs84). Scale bars = 10 μm.

Development • Supplementary information

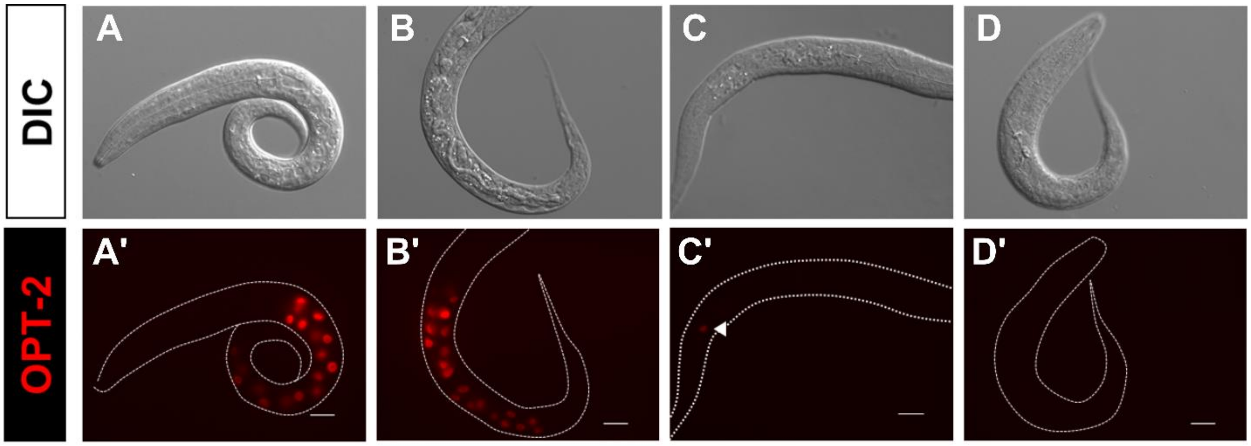


**Fig. S11. Reduced number of differentiated gut cells in differentiation-defective mutants.**

(A-D) DIC images of (A) wildtype, (B) elt-2(-), (C) elt-2(-); elt-7(RNAi), and (D)elt-7(-) end-1(-); elt-2(-) animals. While wildtype and elt-2(-) worms contain a differentiated gut, elt-2(-); elt-7(RNAi) animals lack evident lumen and contains sporadic patches of gut granules. elt-7(-) end-1(-); elt-2(-) triple mutants show no apparent signs of differentiation. (A'-D') Fluorescent images of worms in (A-D) show expression of opt-2p::mCherry. The number of opt-2-expressing cells is markedly reduced in elt-2(-); elt-7(RNAi) (arrowhead). opt-2 expression is completely abolished in elt-7(-) end-1(-); elt-2(-). Scale bars = 10 μm.

Development • Supplementary information

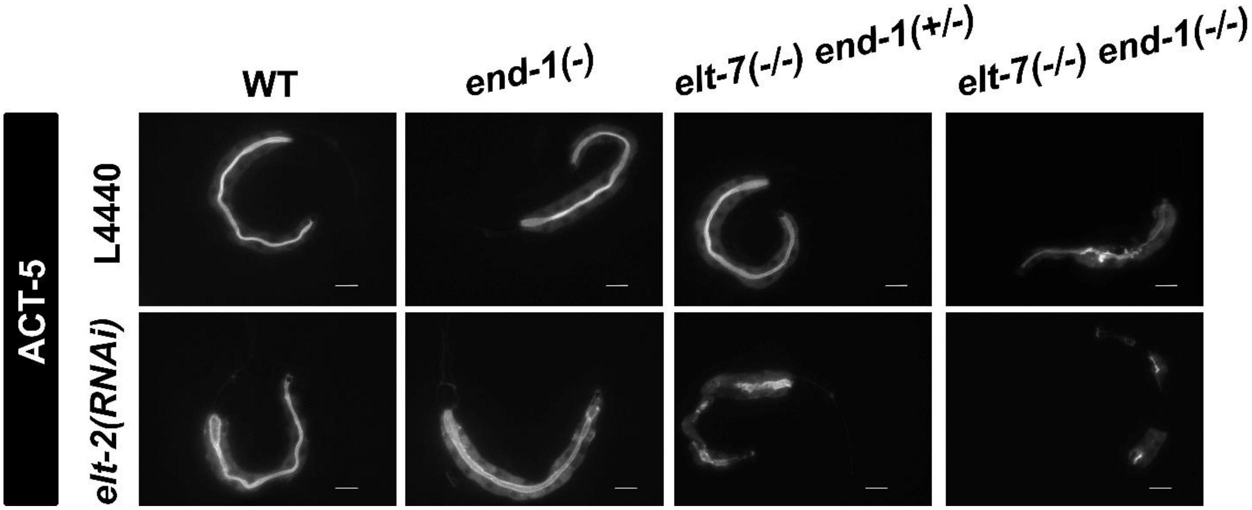


**Fig. S12. END-1, ELT-7, and ELT-2 regulate *act-5* expression.**

act-5 transgene (jyIs13) is expressed strongly in the intestine and weakly in the excretory canal cells. act-5::GFP signals appear sporadic in elt-7(-/-) end-1(+/-); elt-2(RNAi) and elt-7(-/-) end-1(-/-) animals, and are almost completely missing in elt-7(-/-) end-1(-/-); elt-2(RNAi) mutant. The residual act-5 expression in elt-7(-/-) end-1(-/-); elt-2(RNAi) may be due to incomplete RNAi penetrance. Scale bars = 10 μm.

Development • Supplementary information

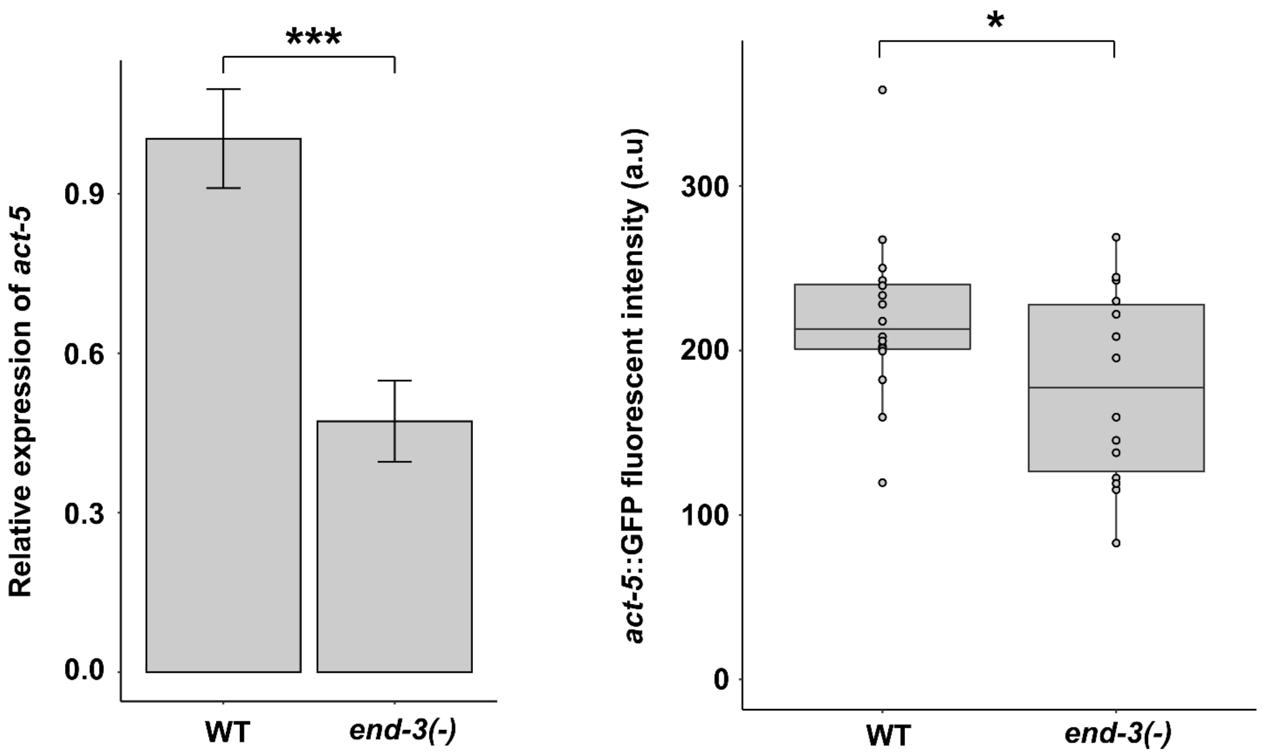


**Fig. S13.** ***act-5*** **is downregulated in** ***end-3(-)*.**

(A, B) act-5 is downregulated in end-3(-) compared to wildtype as measured by (A) RT-qPCR and the (B) expression of act-5::GFP translational reporter (jyIs13). * $p \leq 0.05$, *** $p \leq 0.001$ by two-tail t-test.

Development • Supplementary information

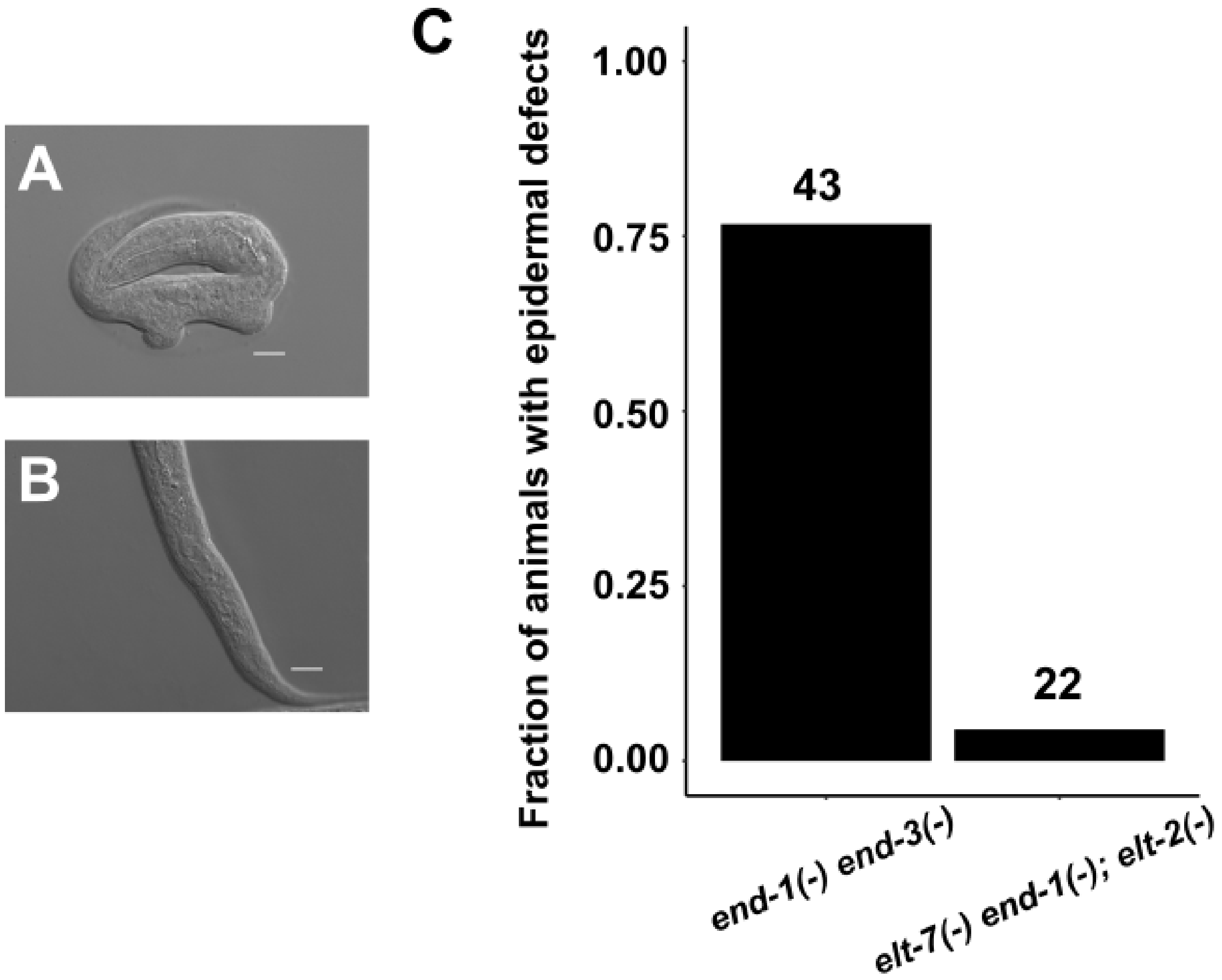


**Fig. S14. Gross epidermal defects in GATA mutants.**

(A) A representative end-1(-) end-3(-) arrested L1 shows gross epidermal defects due to E→C misspecification. (B) elt-7(-) end-1(-); elt-2(-) mutant does not show obvious epidermal defects, as observed by DIC microscopy. Scale bars= 10 µm. (C) A large fraction of end-1(-) end-3(-) mutants showed deformations of the epidermis, which was rarely observed in elt-7(-) end-1(-); elt- 2(-) worms. Number of animals scored for each genotype is indicated.

Development • Supplementary information

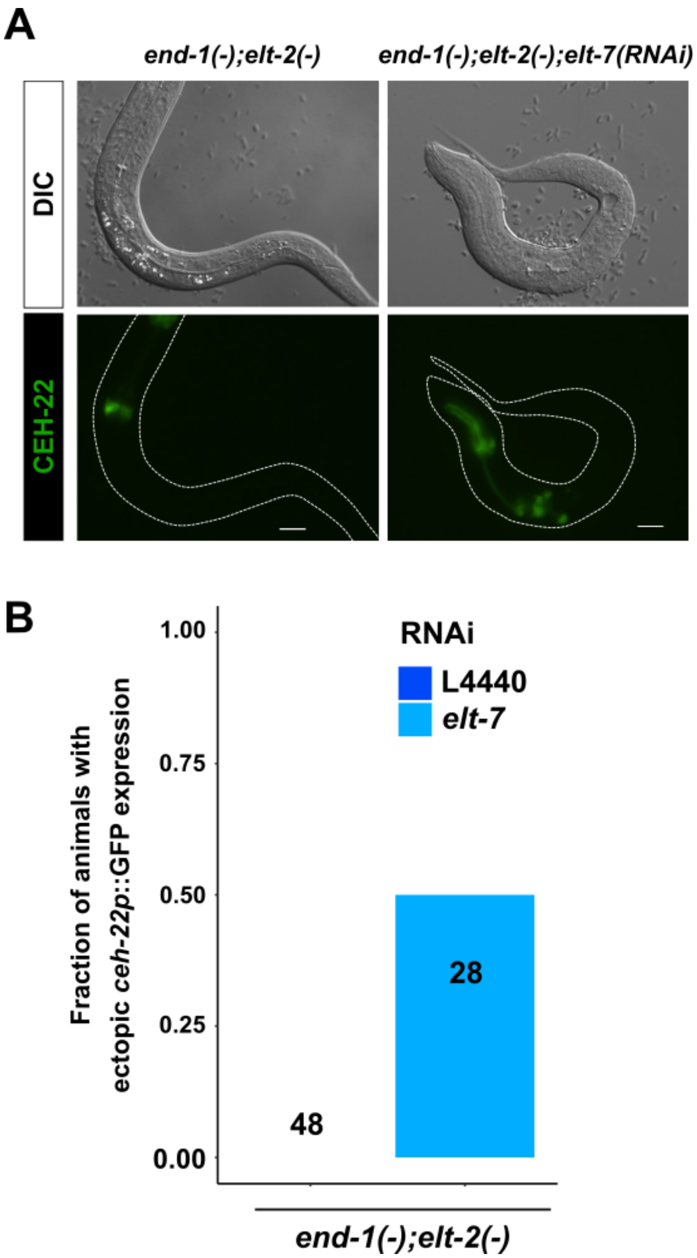


**Fig. S15. Eliminating *end-1*, *elt-7* and *elt-2* causes ectopic expression of *ceh-22* reporter.** **(**A) A representative end-1(-); elt-2(-) worm contains a differentiated gut with defined lumen (top) and wildtype expression pattern of ceh-22 that is restricted to the pharynx (bottom). On the other hand, end-1(-); elt-2(-); elt-7(RNAi) mutant shows no sign of gut differentiation as observed by DIC microscopy (top), and ectopic expression of ceh-22p::GFP reporter (bottom). Scale bars = 10 µm. (B) Knocking out elt-7 inend-1(-); elt-2(-) worms causes ectopic expression of ceh-22p::GFP marker, as shown in (A). Number of animals scored for each genotype is indicated.

Development • Supplementary information

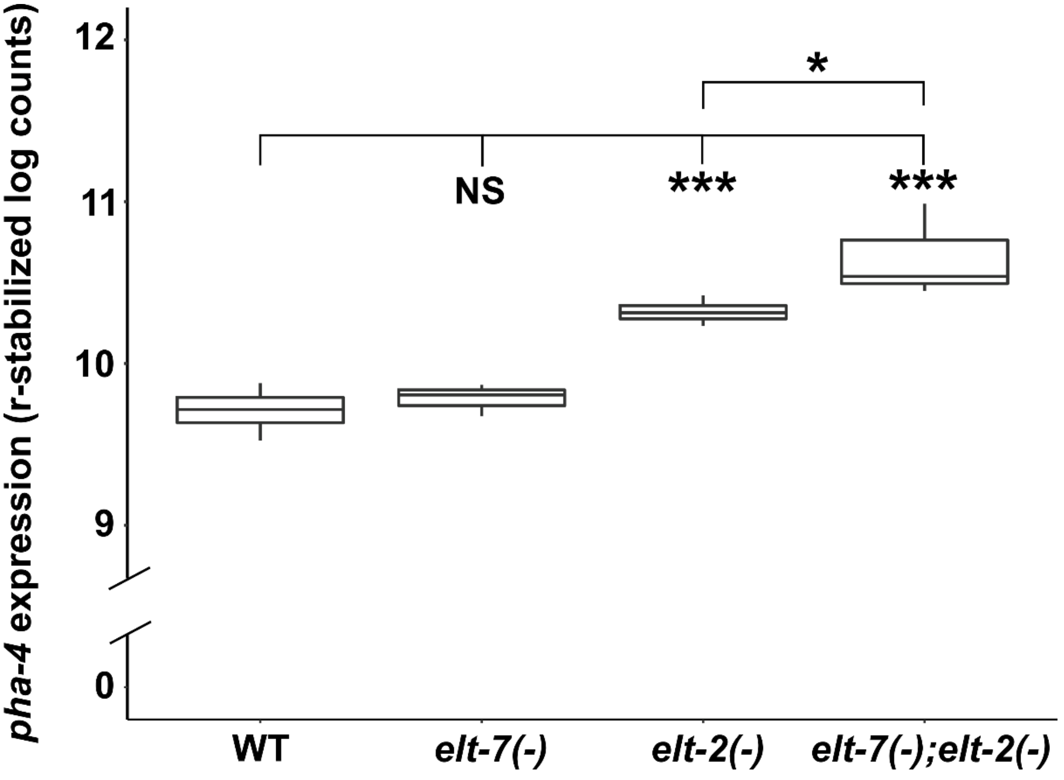


**Fig. S16.** ***pha-4*** **transcript levels are upregulated in** ***elt-2(-)*** **and** ***elt-7(-); elt-2(-).*** Eliminating elt-2 causes upregulation of pha-4 and depleting both elt-7 and elt-2 further enhances this effect. NS $p > 0.05$, * $p \leq 0.05$, *** $p \leq 0.001$ by parametric one-way ANOVA followed by pairwise t-tests with Benjamini & Hochberg correction. RNA-seq data was retrieved from (Dineen et al., 2018).

## Table S1. GATA mutants used in this study

| Gene | Allele | Description |
|---|---|---|
| med-2 | cxTi9744 | Mos1 transposon insertion in the open reading frame, resulting in an early nonsense mutation |
| | ok3199 | Complex substitution (580 bp deletion + 12 bp insertion); removes the entire coding sequence of med-2 |
| med-1 | ok804 | Complex substitution (2527 deletion + 786 bp insertion); removes entire coding sequence of med-1 |
| end-3 | ok1448 | 747 bp deletion; removes exon 2 and 4, including the DNA binding domain |
| end-1 | ok558 | 879 bp deletion; remove exon 3, which includes part of the DNA binding domain |
| | ox134 | 14822 bp deletion; removes the entire end-1 and the neighboring genes ric-7, F58E10.8, and droe-4, which have no known role in endoderm development. |
| elt-7 | tm840 | 616 bp deletion; removes exons 2 and 3, including part of the DNA binding domain |
| elt-2 | ca15 | 2231 bp deletion; removes the entire coding sequence of elt-2 |

Development • Supplementary information

**Table S2. Summary of PCR primers used to detect mutations.**

| Name | Sequence | Short description | Product length (bp) | |
|---|---|---|---|---|
| | | | WT | Mutant |
| EE5<br>EE6 | AGCCCTCAACGTTCCACACGA<br>GCACGTGGGCGTCGGTTTCT | end-1(ox134) forward<br>end-1(ox134) reverse | 757 | 0 |
| EE7<br>EE8 | TGGCATCGTCCTGCCAAGCTC<br>AGGCGGCAAGTGCCATTCGG | elt-7(tm840) forward<br>elt-7(tm840) reverse | 1137 | 518 |
| EE21<br>EE22 | TCAGGCGAGGTGAGACGGGG<br>CGCCTTGGAAAACGTCCGGT | end-1(ok558) forward<br>end-1(ok558) reverse | 1276 | 397 |
| EE25<br>EE26 | CCGGCACAAGATATGACGACAAATTCA<br>TTCCAGCTGCCACAAACATTGCG | end-3(ok1448) forward<br>end-3(ok1448) reverse | 1300 | 553 |
| EE54<br>EE55 | AAATCTCATTATGACAACGAACAAA<br>GCATCCAATCCATGCAATTA | med-2(ok3199) forward<br>med-2(ok3199) reverse | 1204 | 636 |
| EE60<br>EE61 | TCATCACTTTTTGCTGTGGC<br>ATTTCGGCCCTTTTTGTCTC | med-1(ok804) forward<br>med-1(ok804) reverse | 2946 | 1205 |

**Table S3. Worm strains used in this study.** * indicates strain that contains endogenously tagged reporter generated by CRISPR/Cas9.

Click here to download Table S3

**Table S4. Model parameters and outputs of the endoderm GRN.** Expression of each factor is determined by the concentration of its activators multiplied by a coefficient a, representing the strength of the inputs. SKN-1 expression follows a square wave in EMS blastomere. POP-1 expression follows a square wave in the E cell. Feedback coefficients f become nonzero once their respective factors surpass a certain threshold $\Phi$.

Click here to download Table S4

Development • Supplementary information
