# Supplementary material for: Feedforward regulatory logic controls the specification-to-differentiation transition and terminal cell fate during Caenorhabditis elegans endoderm development
Source: Development. 2022 Jun 27;149(12):dev200337. doi: 10.1242/dev.200337 (PMC10656426; doi:10.1242/dev.200337)
Supplement: Supplementary file 1 [file develop-149-200337_review_history.pdf]

Development

Feedforward regulatory logic controls the specification-to-differentiation transition and terminal cell fate during *Caenorhabditis elegans* endoderm development

Chee Kiang Ewe, Erica M. Sommermann, Josh Kenchel, Sagen E. Flowers, Morris F. Maduro, Pradeep M. Joshi and Joel H. Rothman

DOI: 10.1242/dev.200337

Editor: Swathi Arur

Review timeline

Original submission: 10 November 2021
Editorial decision: 12 January 2022
First revision received: 6 April 2022
Editorial decision: 10 May 2022
Second revision received: 10 May 2022
Accepted: 13 May 2022

Original submission

First decision letter

MS ID#: DEVELOP/2021/200337

MS TITLE: Feedforward regulatory logic underlies robustness of the specification-to-differentiation transition and fidelity of terminal cell fate during C. elegans endoderm development

AUTHORS: Chee Kiang Ewe, Erica M Sommermann, Josh Kenchel, Sagen E Flowers, Morris F Maduro, and Joel H Rothman

Thank you for your detailed email. I have carefully reviewed your points listed below. Based on your assertions stated below, I would be happy to revise my decision to ‘Major Revisions’. Please bear in mind that Development only allows one round of revision and the manuscript will be reviewed by the same reviewers who had done so for the first submission. So, it would be extremely important to incorporate each of the points listed below clearly in the manuscript, andprovide a detailed response to the reviewers' comments and highlighting particularly any concerns that have not been included in the revised manuscript.

The revised manuscript and rebuttal will be sent to the original reviewers (if they are still available). If they are convinced by your arguments, then we would be able to consider the manuscript for publication.

To submit a revision, please go to your Author Areaand click on the 'Submit a Revision' link.

Yours sincerely,

Swathi Arur
Handling Editor
Development

The Company of Biologists Ltd
Bidder Building

© 2022. Published by The Company of Biologists under the terms of the Creative Commons Attribution License (https://creativecommons.org/licenses/by/4.0/).

Station Road
Histon, Cambridge, CB24 9LF
Phone:44 (0)1223 632863
Fax:44 (0)1223 632875
dev@biologists.com

The information contained in this message and any attachment is confidential, legally privileged and is intended for the addressee only. Any dissemination, distribution, copying, disclosure or use of this message/attachment or its contents is strictly prohibited and may be unlawful. No contract is intended or implied, unless confirmed by hard copy. If you have received this message in error, please inform the sender and delete it from your mailbox or any other storage mechanism.

The Company Of Biologists Ltd cannot accept liability for any statements made which are clearly the senders' own and not expressly made on behalf of the Company of Biologists Limited or one of their agents.

Reviewer 1

Advance summary and potential significance to field

In this study, Chee Kiang Ewe et al. set out to unpick the regulatory landscape of the endoderm gene regulatory network consisting mainly of GATA TFs and understand the contributions for each member towards the specification and differentiation of endoderm cells. The authors use genetic analysis alongside a computational model to propose that feedforward regulatory logic is important for endoderm specification and differentiation. They propose that END-1 is acting at the interface between specification and differentiation acting together with END-3 and ELT-2/7 in these events respectively. The authors also provide evidence for differentiation factors safeguarding the intestinal cell fate and a properly patterned digestive tract. Understanding the topology and functionality of the endoderm GRN is certainly an interesting problem.
Overall, the study is well-conceived and the manuscript contains interesting findings. My main concern is that the central finding (which is the feed-forward logic in the title) requires further experimental and/or computational support.

Comments for the author

Major Comments
1. While the idea of feedforward biological regulation during gut specification is plausible and consistent with earlier reports, the new evidence provided in this manuscript is only indirect so I felt that strong molecular support is lacking. The authors should provide evidence for transcript levels and onset of expression to support at least some of these feedforward modules (e.g. by quantifying end-3 levels in med-1(-), med-2(-) single mutants and the med-1(-) med-2(-) double mutant). In some cases, the evidence about the shown network topology may be there in the literature, but this is unclear to the reader, so the authors need to make clear what was already known in this area from previous work.
2. The authors argue that “sequential” double mutant combinations are more severely affected than “alternate” double mutants. This is an interesting hypothesis, but the nature of the mutations used is not described anywhere in the manuscript, so it is difficult for the reader to appreciate the significance of this.
Another concern here is that it is difficult to tell throughout the manuscript whether the phenotypic read-out used to assess the fidelity of gut development reflects specification, differentiation or both. The authors discuss at some point how they use epidermal nuclei as an indirect proxy of specification defects, but it is not clear how this might affect their phenotypic interpretation of sequential vs alternate mutants in the first figure (see for example the phenotype of end-3(-); med-1(-) where few worms make it to L1 but many of these animals seem to have a complete lumen – how do the authors interpret this?).
3. The computational model might have been a direction to consolidate the feed-forward logic, but this is not well integrated in the current version of the manuscript and is only little discussed. Did the authors try fitting the data to other predicted network hierarchies and what did they find? How important are the feedforward loops for the model to be robust (as the title claims)? Later in the

© 2022. Published by The Company of Biologists under the terms of the Creative Commons Attribution License (https://creativecommons.org/licenses/by/4.0/).

study, the authors infer new connections in the model based on expanding their genetic analysis (e.g. elt-2 activation by end-3, which is not sequential or alternate)– how do these additions affect the performance of their model?
4. It is always nice when computational models lead to some new testable predictions. The model seems to predict the onset and levels of elt-2 expression in single/double mutants, but there is no follow up experiment in any background to validate these predictions for example using an elt-2 reporter or in situ.
5. The authors suggest the END-1 plays a key role acting at the transition between specification and differentiation. However, it is not shown whether this function is specific to END-1 or whether END-3 may also play a similar role. Have the authors tested the phenotypic effect of the end-3(-);elt-7(-); elt-2(-) triple mutant or differentiation markers (e.g. act-5) in end-3(-) mutants?

Other Comments:
Throughout the paper, it is unclear whether the transcription factor interactions described are likely to be direct are not. The authors should discuss this point based on available ChipSeq data and the previous literature.
Fig. 2L: Elt-2(-) and elt-7(-) controls or end-3(-) mutants would help the reader appreciate the significance of this observation.
Fig. S4B Please annotate images to help readers see where the intestinal cells are.
Line 246-248 Check grammar
Line 253 Strongly forward-driven sounds a bit odd
Line 259 Pop-1 dependent activation of end-1 and end-3 (and elsewhere in the manuscript) needs better introduction for the reader to understand.
Line 268-271 This sentence is unclear – what are the experimental outcomes you refer to ?

Reviewer 2

Advance summary and potential significance to field

This manuscript aimed to decipher the functional redundancies and interactions between the GATA transcription factors for faithful gut development in C. elegans. Using a combination of single/double/triple genetic mutants or RNAi they found that removal of a single factor or alternate factors in the cascade results in a much milder effect on endoderm development and gut differentiation than elimination of any two factors that are sequentially expressed factors within the regulatory cascade, which appears to be upheld by the modeling analysis. Consequently, they propose a feedforward regulatory logic for the gut development. They further claimed that END-1 mediates gut specification-to-differentiation. They finally showed the roles for key GATA factors in establishing spatial regulatory state domains by acting as transcriptional repressors that appear to define the boundaries of the digestive tract. The genetic work has been beatifically done.
The regulatory control by GRN is definitely an exciting area that is worthy of attention.

Comments for the author

This manuscript aimed to decipher the functional redundancies and interactions between the GATA transcription factors for faithful gut development in *C. elegans*. Using a combination of single/double/triple genetic mutants or RNAi, they found that removal of a single factor or alternate factors in the cascade results in a much milder effect on endoderm development and gut differentiation than elimination of any two factors that are sequentially expressed factors within the regulatory cascade, which appears to be upheld by the modeling analysis. Consequently, they propose a feedforward regulatory logic for the gut development. They further claimed that END-1 mediates gut specification-to-differentiation. They finally showed the roles for key GATA factors in establishing spatial regulatory state domains by acting as transcriptional repressors that appear to define the boundaries of the digestive tract. The genetic work has been beatifically done. The regulatory control by GRN is definitely an exciting area that is worthy of attention. However, there are several concerns/comments regarding their claims.

© 2022. Published by The Company of Biologists under the terms of the Creative Commons Attribution License (https://creativecommons.org/licenses/by/4.0/).

1. Their conclusions are mainly based on the expression onset of various GATA factors that were previously defined using various techniques. Although some of these expression onsets are obvious, while others are less convincing. Most these GFP expression patterns were obtained in the transgenic lines generated through array insertion if I am correct (no details can be found in the manuscript and no discussion of their complications was given). These transgenes often suffer from uncertainty in copy number variation, transgene insertion site as well as incomplete regulatory sequences included, making them less convincing in deducing expression onset. The single cell RNA-seq data show little correspondence in terms of expression onset and dynamics. A better way of defining expression dynamics is by antibody staining with proper internal control, or through in-frame knock-in of a single copy of GFP or other fluorescence markers at the 5' or the 3' of the target gene. This is particularly relevant to the expression onset of MEDs.

2. END-1 and 3 were reported previously to regulatory different target genes as long as cellular behaviors during embryogenesis. The functional redundancy of the two may gain more insight by comparing their target genes, which could be overlapping but different in some ways. Time-series ChIP-seq analysis were performed for almost all the TFs in C. elegans. Exploration of these data would potentially help understand the missing links from their analysis in terms of regulatory redundancy.

3. Figure 3: ELT-2 antagonizes end-1 expression. These evidences are weak. 4E and 8E last a long period. Timing of imaging is critical. Data acquired using mutant with precisely timed imaging would be much more convincing.

4. Figure 4: ELT-2 and ELT-7 repress pharyngeal fate in the intestine. According to their definition, the role of ELT-2 and ELT-7, i.e., repressing pharyngeal fate in the intestine, should classify them as specifier rather than differentiator? This is contradictory to their initial definition of the TFs' roles. A clear definition of specification and differentiation is required early in the manuscript regarding what they meant by gut specification and differentiation.

5. More details are needed in the Methods part. For example, how they genotype the double/triple mutant of a dead animal? How to distinguish hetero versus homozygote for each allele in a single dead/dying animal? Also needed are the details of the transgenic animals, strain names, genotypes, methods of transgenesis.

Specific comments:

1. Line 124, Typo. "performed at room temperature (20-23OC)"
2. Line 968 "Expression of MED-2 and MED-1 protein-fusion reporters in staged embryos." "Supplementary Figure 4: The endoderm GATA factors are deployed in temporal order." How were the transgenes made?
3. Line 422 "end-3(-) double mutant animals, elt-7(-) end-1(-); elt-2(-) triple mutant larvae contain a wildtype." This is not a triple mutant.
4. Fig. 4D X axis labeling is confusing.
5. Line 488 "(Köppen et al., 2001; Sommermann et al., 2010). However, while end-1(-) and elt-7(-/-) end-" meaning end-1(-/-)?
6. Line 488 "However, while end-1(-) and elt-7(-/-) end-1(+/-) animals show wildtype ajm-1 expression pattern, ajm-1 signal is markedly elevated in the anterior intestinal terminus of elt-7(-/-) end-1(-/-) animals (Figure 5B-E)." What about the ajm-1 signal in elt-2(-/-) end-1(-/-) animals? These data would be important for their claim of sequential regulation.
7. The sequential relationship between med-1 and med-2 seems not known until this manuscript, but it is assumed to be established in Figure 1 (med-2 -> med-1) before showing the evidence in the Results (line: 304): Variation in temporal expression explains distinct functions for MED-1 and -2. This organization confusing. I suggest the authors should at least mention that before showing the Figure 1 or discuss the sequential activation of med-1 and -2 eariler.
8. Figure 1I: Does the X-axis closer to "0" means later expression of elt-2?
9. Line 573: "Interestingly".

© 2022. Published by The Company of Biologists under the terms of the Creative Commons Attribution License (https://creativecommons.org/licenses/by/4.0/).

10. Line 664 (Figure 6): “end-1(-)”.

Optional points to address:

1. According to line 495-497 and Figure 5L, ELT-2 is expressed at wildtype levels in elt-7(-/-) end-1(-/-) larvae, but this mutant shows 0% of complete lumen in L1 (Figure 1A). Wondering if ELT-2 expression is delayed in elt-7(-/-) end-1(-/-) mutants during embryogenesis that leads to the missed differentiation even if the expression seems normal in larvae.
2. In Figure 6, 3 genes are involved in activation of terminal differentiation genes, which are END-1, ELT-7, and ELT-2. While in line 234-237 and Figure 1D and G, elt-7(-) end-3(-) mutants (with suboptimal expression of end-1), 61% of L1 has complete lumen. It seems that ELT-2 alone is not enough for the proper gut differentiation to have a complete lumen, while elt-7(-) end-3(-) mutant has a partial end-1 to help with the ELT-2 for terminal differentiation, probably explaining the higher ratio of complete lumen. However, end-1(ok558); elt-2(-) showed about 90% of complete lumen in L1 (Figure 1A), leading to a possibility that ELT-7 is the most important TF for terminal differentiation. However, this seems to contradict with the impression that ELT-2 is the most important TF for gut differentiation. Any thought on this observation.

Reviewer 3

Advance summary and potential significance to field

The authors describe the gene regulatory networks (GRN) that underlies the specification and differentiation of the endoderm, which gives rise to the intestine in C. elegans. The development of the C. elegans intestine is orchestrated through the activity of previously described GATA-type transcription factors, which are sequentially expressed and active. The authors combined different mutant backgrounds creating double / triple mutants and based on the observed mutant phenotypes they conclude that the transcription factors are acting in a recursive series of interlocked feedforward modules.
Overall, the manuscript is well written. The presented findings and derived conclusions can be considered as a refinement of many previously known aspects of C. elegans intestinal development.

Comments for the author

The authors describe in their manuscript ‘Feedforward regulatory logic underlies robustness of the specification-to-differentiation transition and fidelity of terminal cell fate during C. elegans endoderm development’ the gene regulatory networks (GRN) that underlies the specification and differentiation of the endoderm, which gives rise to the intestine in C. elegans. The development of the C. elegans intestine is orchestrated through the activity of previously described GATA-type transcription factors, which are sequentially expressed and active. The authors combined different mutant backgrounds creating double / triple mutants and based on the observed mutant phenotypes they conclude that the transcription factors are acting in a recursive series of interlocked feedforward modules.
Overall, the manuscript is well written. The presented findings and derived conclusions can be considered as a refinement of many previously known aspects of C. elegans intestinal development.

Major Concerns:
While the overall assessment of the feedforward loops is rather a refinement of previously described transcription factor actions that are relevant for gut development, some observations point towards new findings – yet the investigations are incomplete:
1. The observation that end-1 becomes depressed in early embryos when ELT-2/7 are depleted has not been described before. Unfortunately, the authors only argue that the effect is rather weak and provide no further experiments such using mutants to support the RNAi experiments (see also comment 3 below). This could have provided stronger effects.
2. Overall, the upregulation of pha-4 upon depletion of ELT-2 in the intestine is probably the most relevant finding in this study. The proposed ‘dual role’ of ELT-2 with regard to regulating pha-4 expression would be clear conceptual advancement. While ectopic pha-4 induction in the anterior gut in elt-7(-); elt-2(-) and elt-7(-) end-1(-); elt-2(-) mutants supports the transgene-based results for ectopic myo-2::gfp and ceh-22::gfp induction in the intestine, these effects need better

© 2022. Published by The Company of Biologists under the terms of the Creative Commons Attribution License (https://creativecommons.org/licenses/by/4.0/).

experimental validation. All data concerning this important aspect are based on gfp-reporter transgenes! Such transgenes do not always reflect endogenous gene expression, in particular in modified genetic backgrounds. It is highly important to support the observations based on the gfp-reporter transgenes by assessing endogenous gene expression. smFISH is feasible and straightforward as this approach would not require generating new strains.
3. It is not clear why in some cases elt-2(-) combined with elt-7 RNAi is being used (e.g. when scoring opt-2p::mCherry) although in several other cases (e.g. when scoring myo-2p::GFP) the double mutant elt-2(-); elt-7(-) has been used. This is somewhat inconsistent and using mutant alleles to support RNAi results should be used whenever possible.

Minor concerns:
4. Knowing that the authors are experts and experienced with identifying in a proper way intestinal tissue it would be good to provide some evidence that the ectopic signals for myo-2, ceh-22, pha-4 are indeed in intestinal cells.
5. The title is too long!
6. line 84 (page5): '…to a clone 20 cells…' – seems like 'of' is missing
7. Authors provide no idea how repression of end-1 by ELT-2/-7 could take place at the molecular level.

First revision

<u>Author response to reviewers' comments</u>

We are grateful to both you and the reviewers for very thoughtful and helpful comments on our manuscript. We have revised the manuscript extensively in response to the reviews, as we describe below. This includes addition of many new findings that resulted in modification of 5 figures (Figure 1, 4, 6; Supplementary Figure 1, 7) and an additional 7 new figures (Supplementary Figure 2, 5, 6, 8, 10, 13, 16), all of which respond to all concerns of the reviewers. We believe that these extensive revisions effectively address all of the issues raised by the reviewers and that the revised manuscript is a much stronger paper. Thank you for considering this revised manuscript for publication in *Development*.

Below, the reviewers' comments are indicated in italics and our responses, including where revisions are made, follow each comment.

Reviewer 1 Advance Summary and Potential Significance to Field:

In this study, Chee Kiang Ewe et al. set out to unpick the regulatory landscape of the endoderm gene regulatory network consisting mainly of GATA TFs and understand the contributions for each member towards the specification and differentiation of endoderm cells. The authors use genetic analysis alongside a computational model to propose that feedforward regulatory logic is important for endoderm specification and differentiation. They propose that END-1 is acting at the interface between specification and differentiation acting together with END-3 and ELT-2/7 in these events respectively. The authors also provide evidence for differentiation factors safeguarding the intestinal cell fate and a properly patterned digestive tract. Understanding the topology and functionality of the endoderm GRN is certainly an interesting problem. Overall, the study is well-conceived and the manuscript contains interesting findings. My main concern is that the central finding (which is the feed-forward logic in the title) requires further experimental and/or computational support.

Reviewer 1 Comments for the Author:

Major Comments

6. While the idea of feedforward biological regulation during gut specification is plausible and consistent with earlier reports, the new evidence provided in this manuscript is only indirect so I felt that strong molecular support is lacking. The authors should provide evidence for transcript

© 2022. Published by The Company of Biologists under the terms of the Creative Commons Attribution License (https://creativecommons.org/licenses/by/4.0/).

levels and onset of expression to support at least some of these feedforward modules (e.g. by quantifying end-3 levels in med-1(-), med-2(-) single mutants and the med-1(-) med-2(-) double mutant). In some cases, the evidence about the shown network topology may be there in the literature, but this is unclear to the reader, so the authors need to make clear what was already known in this area from previous work.

We thank the reviewer for these thoughtful comments. Transcriptomic analyses of early embryos have shown sequential expression of the GATA genes, as we have now highlighted in Supplementary Figure 2; however, these data showed only that expression is sequential and do not reveal the regulatory logic *per se*. Our computer modeling approach, which we have now expanded substantially (see below), coupled with our extensive genetic analysis, strongly support the recursive feedforward network architecture of the gene regulatory network for endoderm.

As requested by the reviewer and now reported in the revised manuscript, we have measured *end-3* expression levels using a CRISPR-tagged endogenous reporter and found that knocking out *med-1* or *med-2* alone does not significantly alter END-3 expression (Supplementary Figure 5), In contrast, Maduro MF, et al. (2015) showed that eliminating both *med-1* and *med-2* abolishes the expression of a full-length (transgene) *end-3* reporter, implying that MED-1 and MED-2 function redundantly to activate *end-3*, as is also revealed by our genetic analyses (Figure 1; see lines 280-295).

7. The authors argue that “sequential” double mutant combinations are more severely affected than “alternate” double mutants. This is an interesting hypothesis, but the nature of the mutations used is not described anywhere in the manuscript, so it is difficult for the reader to appreciate the significance of this.

We regret excluding this critically important information from the original manuscript. We have now incorporated Supplementary Table 1, which describes the nature of all mutations used in this study. It is important to note that all mutations used in the study are null mutations.

Another concern here is that it is difficult to tell throughout the manuscript whether the phenotypic read-out used to assess the fidelity of gut development reflects specification, differentiation or both. The authors discuss at some point how they use epidermal nuclei as an indirect proxy of specification defects, but it is not clear how this might affect their phenotypic interpretation of sequential vs alternate mutants in the first figure (see for example the phenotype of end-3(-); med- 1(-) where few worms make it to L1 but many of these animals seem to have a complete lumen
- how do the authors interpret this?).

Throughout the entire revised manuscript, we have now clearly indicated whether the gut defects we observed in the mutants reflect loss of specification or differentiation (see lines 200-206, 222-235, 241-256, 263-272, 447-464, 472-486). In sum, removing the functions of fate-specifying factors, for example, in the *med-2(-); med-1(-)* double mutant, the *end-3(-); med-1(-)* double mutant, and the *end-1(-) end-3(-)* double mutant causes endoderm precursors to undergo wholesale transformation into the mesectodermal C fate, indicating their roles in specification. This cell fate transformation generally leads to gastrulation defects and severe embryonic lethality as we report in Figure 1C. In contrast, we observe no evidence for such cell fate transformation in the *elt-7(-) end-1(-)* double, the *elt-7(-); elt-2(-)* double, or the *elt-7(-) end-1(-); elt-2(-)* triple mutant, implicating their roles in post-specification abrogation of differentiation (and underscoring the role for END-1 in both processes).

The *end-3(-); med-1(-)* double mutant exhibits severe embryonic lethality owing to diminished *end-1* expression and an E→C cell fate transformation, as highlighted in Figure 1F in the revised manuscript. However, as this reviewer pointed out, of the hatched L1s, many contain a complete lumen. This is not unexpected as maternal POP-1 is apparently sufficient to activate *end-1* above a threshold level (see, for example, Raj A. et al., 2010) that activates normal endoderm specification and differentiation in a minor fraction of embryos. We have clarified this point in lines 253-256 of the revised manuscript.

8. The computational model might have been a direction to consolidate the feed-forward logic, but this is not well integrated in the current version of the manuscript and is only little

© 2022. Published by The Company of Biologists under the terms of the Creative Commons Attribution License (https://creativecommons.org/licenses/by/4.0/).

discussed. Did the authors try fitting the data to other predicted network hierarchies and what did they find?

We have now substantially expanded the application and discussion of the computational model and highlighted the modeling approach to studying GRN topology in the revised manuscript (see lines 296-328). We have extended our analysis using the original tier model, in which 2 redundant MEDs → 2 redundant ENDs → 2 redundant ELTs and found that it fits poorly with the transcriptomic data, despite iterating the fitting algorithm for the same number of cycles (~200,000) as with the feedforward model (final sum-of-squares of the tier model = 2695). In contrast, the expression data is much better fitted by the feedforward model (final sum-of-squares of the feedforward model = 1298). Moreover, compared with the feedforward model (Figure 1K, K'), the predicted *elt-2* expression in the tier model does not correlate as well with measured viability of the mutant combinations (Figure 1J, J'). Thus, the combination of our experimental analysis of mutation combinations and our computational modeling strongly support a feedforward, rather than a tiered architecture for the endoderm GRN.

How important are the feedforward loops for the model to be robust (as the title claims)?

We proposed that the feedforward regulatory logic may give rise to perdurant transcriptional response after the initial inputs are switched off, as suggested previously by mathematical modeling (see lines 631-634 in the revised manuscript) (Mangan S. et al., 2003). Although this architecture could serve to provide an important buffering mechanism against genetic and environmental variation, we will not make such a claim as we do not have direct evidence for such a hypothesis (which would be very challenging to obtain). We have therefore modified the title, including removal of "robust", to: "*Feedforward regulatory logic controls the specification-to-differentiation transition and terminal cell fate during* C. elegans *endoderm development*."

Later in the study, the authors infer new connections in the model based on expanding their genetic analysis (e.g. elt-2 activation by end-3, which is not sequential or alternate)- how do these additions affect the performance of their model?

We are grateful for the reviewer's question and implied suggestion. For the revision, we performed the suggested analysis and found that it resulted in <u>increased</u> support from the model. Indeed, as reported in the revised manuscript, we found that the addition of an *end-3* → *elt-2* relationship in the computational model <u>improves</u> its fit to the transcriptomic data, with a better sum-of-squares of 1298, compared to the previous feedforward model without *end-3* → *elt-2* that showed a sum-of-squares of 1524.

9. It is always nice when computational models lead to some new testable predictions. The model seems to predict the onset and levels of elt-2 expression in single/double mutants, but there is no follow up experiment in any background to validate these predictions for example using an elt-2 reporter or in situ.

We agree with the importance of validating our computational model. In the revised manuscript, therefore, we now report the expression of a CRISPR-tagged endogenous ELT-2 reporter in selected mutant strains to test the model predictions. We found that ELT-2 expression in these mutant combinations is <u>strongly</u> positively correlated with the predicted levels (Spearman rho = 1.00, p = 0.017), <u>demonstrating the high performance and predictive power of the computational model</u> (Supplementary Figure 6). We have modified the text to report this additional finding and validation of the model (lines 315-320).

10. The authors suggest the END-1 plays a key role acting at the transition between specification and differentiation. However, it is not shown whether this function is specific to END-1 or whether END-3 may also play a similar role. Have the authors tested the phenotypic effect of the end-3 (-);elt-7(-); elt-2(-) triple mutant or differentiation markers (e.g. act-5) in end-3(-) mutants?

We thank the reviewer for the comments and suggestions. We now report in the revised manuscript that *end-3(-); elt-2(-); elt-7(RNAi)* animals show a similar phenotype to that of *end-1(-); elt-2(-); elt-7(RNAi)* animals, with little sign of gut differentiation as reported in the new Supplementary Figure 10. Further, we now also report that *act-5* is downregulated in *end-3(-)*

© 2022. Published by The Company of Biologists under the terms of the Creative Commons Attribution License (https://creativecommons.org/licenses/by/4.0/).

mutants based both on quantitative RT-PCR and on *act-5* transgene reporter expression (Supplementary Figure 13). However, it is unclear whether these effects are direct or reflect reduction of *end-1* expression, which is known to be downregulated in the absence of *end-3* (Maduro et al., 2007; Raj et al., 2010). As it is difficult to distinguish the direct contributions of *end-3* from that of its target *end-1*, we have chosen to focus primarily on END-1 in this study (see lines 384-393).

Other Comments:

Throughout the paper, it is unclear whether the transcription factor interactions described are likely to be direct are not. The authors should discuss this point based on available ChipSeq data and the previous literature.

We agree that studying the regulatory interaction between transcription factors and their target genes would be invaluable for the understanding of gene regulatory network. Unfortunately, however, ChIP-seq data are not available for the GATA factors at the necessary early embryonic stages for this study. Please see also the comments from reviewer 2 point #2.

Fig. 2L: Elt-2(-) and elt-7(-) controls or end-3(-) mutants would help the reader appreciate the significance of this observation.

We have now included a figure in the revised manuscript showing *act-5* expression in *elt-7(-)*, *elt-2(-)* double mutants and *elt-7(-); elt-2(-)* double mutants, as obtained from the RNA-seq data of Dineen A. et al (2017) (new Supplementary Figure 8). We also now report that *act-5* is downregulated in *end-3(-)* mutants (new Supplementary Figure 13).

Fig. S4B Please annotate images to help readers see where the intestinal cells are.

We have removed this figure from the revised manuscript.

Line 246-248 Check grammar

This has been corrected in the revision.

Line 253 Strongly forward-driven sounds a bit odd

We have deleted “strongly”.

Line 259 Pop-1 dependent activation of end-1 and end-3 (and elsewhere in the manuscript) needs better introduction for the reader to understand.

We have now modified the Introduction to expand the discussion of POP-1 (see lines 87-97). We have also included a new panel to Supplementary Figure 1 to demonstrate the nucleocytoplasmic distribution of POP-1 in response to Wnt/MAPK/Src in the E blastomere (also see lines 996-1004)

Line 268-271 This sentence is unclear - what are the experimental outcomes you refer to?

We have changed the sentence to make it clear that we are referring to the severe developmental defects of the mutant combinations (lines 320-326).

Reviewer 2 Comments for the Author:

This manuscript aimed to decipher the functional redundancies and interactions between the GATA transcription factors for faithful gut development in C. elegans. Using a combination of single/double/triple genetic mutants or RNAi, they found that removal of a single factor or alternate factors in the cascade results in a much milder effect on endoderm development and gut differentiation than elimination of any two factors that are sequentially expressed factors within the regulatory cascade, which appears to be upheld by the modeling analysis. Consequently, they propose a feedforward regulatory logic for the gut development. They further claimed that END- 1 mediates gut specification-to-differentiation. They finally showed the roles for key GATA

© 2022. Published by The Company of Biologists under the terms of the Creative Commons Attribution License (https://creativecommons.org/licenses/by/4.0/).

factors in establishing spatial regulatory state domains by acting as transcriptional repressors that appear to define the boundaries of the digestive tract. The genetic work has been beatifically done. The regulatory control by GRN is definitely an exciting area that is worthy of attention. However, there are several concerns/comments regarding their claims.

10. Their conclusions are mainly based on the expression onset of various GATA factors that were previously defined using various techniques. Although some of these expression onsets are obvious, while others are less convincing. Most these GFP expression patterns were obtained in the transgenic lines generated through array insertion if I am correct (no details can be found in the manuscript and no discussion of their complications was given). These transgenes often suffer from uncertainty in copy number variation, transgene insertion site as well as incomplete regulatory sequences included, making them less convincing in deducing expression onset. The single cell RNA-seq data show little correspondence in terms of expression onset and dynamics. A better way of defining expression dynamics is by antibody staining with proper internal control, or through in-frame knock-in of a single copy of GFP or other fluorescence markers at the 5' or the 3' of the target gene. This is particularly relevant to the expression onset of MEDs.
We regret that the original manuscript did not include the vital information that <u>all critical experiments were performed by analyzing the endogenous genes (not transgenes)</u> obtained by CRISPR modification of the normal locus, which does not detectably interfere with their normal function. We fully agree that transgenes with varying copy number and uncharacterized insertion sites can produce artifactual results, which is precisely why we chose to use CRISPR-generated tagging of the endogenous genes for these experiments (Figure 3; modified Figure 4G-I; Figure 5I, J; new Supplementary Figure 5; new Supplementary Figure 6A, B). We have modified the text in the revised manuscript accordingly to highlight the nature of the reporters used and to make it clear that these results are based on endogenous gene expression. In the revision, we have also highlighted the strains carrying endogenously tagged reporters in Supplementary File 1.

An exception is that we used integrated arrays to monitor the dynamics of *med-1* and *med-2*. Because of the high degree of similarity (98% identity) of their coding and flanking sequences, smFISH experiments and CRISPR-mediated knock-in of reporters are particularly challenging. Additionally, as the protein sequences are virtually identical, it is not possible to obtain isoform-specific antibodies. However, although the experiments may well reflect the true expression of these genes, we have removed the analysis of GFP::MED-1 and MED-2::GFP fusion proteins from the revised manuscript as a result of the concerns about integrated arrays. This change has no impact on our major conclusions.

11. END-1 and 3 were reported previously to regulatory different target genes as long as cellular behaviors during embryogenesis. The functional redundancy of the two may gain more insight by comparing their target genes, which could be overlapping but different in some ways. Time-series ChIP-seq analysis were performed for almost all the TFs in C. elegans. Exploration of these data would potentially help understand the missing links from their analysis in terms of regulatory redundancy.

We fully agree that studying the regulatory interactions between transcription factors and their target genes will be invaluable for further illuminating the gene regulatory network. However, unfortunately, END-1 and END-3 ChIP-seq studies have not been performed (http://epic.gs.washington.edu/modERN/). Furthermore, the very short time window of END-1 and END-3 is barely captured by systematic ChIP-seq type of studies such as by MODencode. Indeed, despite a demonstration that GFP-tagged MED-1 binds to the *end-1* and *end-3* promoters *in vivo* (by transgene spot assays; Maduro M, et al., 2002) and *in vitro* (by gel shift assays; Broitman-Maduro G. et al., 2005), the MODencode data for MED-1 contain only artifactual targets.

12. Figure 3: ELT-2 antagonizes end-1 expression. These evidences are weak. 4E and 8E last a long period. Timing of imaging is critical. Data acquired using mutant with precisely timed imaging would be much more convincing.

We agree with the reviewer that experiments with the *elt-2* chromosomal mutation would bolster our results. However, examining *elt-2(-)* early embryos is not straightforward as the *elt-2(+)* rescuing array we used to balance the mutation contains *unc-119::GFP*, whose expression is undetectable until mid-embryonic (comma/1.5-fold) stages. In the revised manuscript, we have

© 2022. Published by The Company of Biologists under the terms of the Creative Commons Attribution License (https://creativecommons.org/licenses/by/4.0/).

added text to clearly acknowledge the potential caveats and, while the effect is significant, we have substantially toned down our claim of ELT-2/7 repression of *end-1* expression (lines 394- 406, 664-449).

13. Figure 4: ELT-2 and ELT-7 repress pharyngeal fate in the intestine. According to their definition, the role of ELT-2 and ELT-7, i.e., repressing pharyngeal fate in the intestine, should classify them as specifier rather than differentiator? This is contradictory to their initial definition of the TFs' roles. A clear definition of specification and differentiation is required early in the manuscript regarding what they meant by gut specification and differentiation.

We thank the reviewer for the comments and suggestions, which is similar to comment #2 by reviewer 1 (see above). As noted, in the revised manuscript, we have clearly defined specification and differentiation in the Introduction (lines 48-54) and have now clearly indicated whether the gut defects we observed in the mutants reflect disruption of specification or instead differentiation (see lines 200-206, 222-235, 241-256, 263-272, 447-464, 472-486). For more details, see reviewer 1 comment #2 response.
Although we found that pharyngeal genes are ectopically expressed in *elt-7(-); elt-2(-)* double mutants, the evidence suggests that this effect is not the result of E→MS misspecification, as we now describe in the revised manuscript (lines 472-486). Gut cells in *elt-7(-); elt-2(-)* double mutants do not appear to fully adopt the pharyngeal cell fate; rather, upregulation of PHA-4 leads to aberrant expression of pharyngeal genes in the cells that fail to maintain a terminal differentiated state post-specification.

14. More details are needed in the Methods part. For example, how they genotype the double/triple mutant of a dead animal? How to distinguish hetero versus homozygote for each allele in a single dead/dying animal?

We have included the required details in the Methods section of the revised manuscript (see lines 125-136). Mutations and transgenes were validated by PCR and sequencing. We now provide the primer sequences in the new Supplementary Table 2.

Also needed are the details of the transgenic animals, strain names, genotypes, methods of transgenesis.

This information is now included in the new Supplementary File 1, which contains all strain names, genotypes, and sources of the strains. The strains that carry CRISPR-tagged endogenous reporters are highlighted.

Specific comments:

3. Line 124, Typo. "performed at room temperature (20-23OC)"

Corrected in the revision.

4. Line 968 "Expression of MED-2 and MED-1 protein-fusion reporters in staged embryos." "Supplementary Figure 4: The endoderm GATA factors are deployed in temporal order." How were the transgenes made?

We had used published strains carrying the integrated arrays for these experiments (Maduro MF., et al., 2001). As noted above in response to reviewer 1, we recognize that the arrays may not reflect endogenous expression and have therefore removed the sections discussing the temporal deployment of *med-2* and *med-1* in the revised manuscript, with no substantive impact on our major conclusions.

5. Line 422 "end-3(-) double mutant animals, elt-7(-) end-1(-); elt-2(-) triple mutant larvae contain a wildtype." This is not a triple mutant.

We have corrected the text. We used *end-1(-); elt-2(-); elt-7(RNAi)* animals for these experiments.

© 2022. Published by The Company of Biologists under the terms of the Creative Commons Attribution License (https://creativecommons.org/licenses/by/4.0/).

6. Fig. 4D X axis labeling is confusing.

The figure has been modified to correct this deficiency and the X axis parameters are now clearly discerned.

7. Line 488 "(Köppen et al., 2001; Sommermann et al., 2010). However, while end-1(-) and elt-7(-/-) end-" meaning end-1(-/-)?

The reviewer is correct. We modified the text to clarify this point.

8. Line 488 "However, while end-1(-) and elt-7(-/-) end-1(+/-) animals show wildtype ajm-1 expression pattern, ajm-1 signal is markedly elevated in the anterior intestinal terminus of elt-7(- /-) end-1(-/-) animals (Figure 5B-E). "What about the ajm-1 signal in elt-2(-/-) end-1(-/-) animals? These data would be important for their claim of sequential regulation.

We do not believe this experiment would further illuminate our findings or conclusions because we have previously observed similar aberrant expression of *ajm-1* in *elt-2(-)* single mutant (Sommermann EM., et al, 2010). Our data indicate that END-1 and ELT-7 function synergistically to represses valve-like characteristics in the midgut, independent of ELT-2 functions. These results do not reflect sequential regulation.

9. The sequential relationship between med-1 and med-2 seems not known until this manuscript, but it is assumed to be established in Figure 1 (med-2 -> med-1) before showing the evidence in the Results (line: 304): Variation in temporal expression explains distinct functions for MED-1 and -2. This organization confusing. I suggest the authors should at least mention that before showing the Figure 1 or discuss the sequential activation of med-1 and -2 eariler.

We thank the reviewer for the suggestions. We have now incorporated the discussion of MED-2 and MED-1 function into the first section of the Results (see lines 280-295). We have also removed the section on temporal expression of MEDs as discussed above.

10. Figure 1I: Does the X-axis closer to "0" means later expression of elt-2?

Yes, this is correct as we have now clarified in the main text and figure legend (lines 316-322, 363-364)

11. Line 573: "Interestingly".

Corrected.

10. Line 664 (Figure 6): "end-1(-)".

Corrected.

Optional points to address:

1. According to line 495-497 and Figure 5L, ELT-2 is expressed at wildtype levels in elt-7(-/-) end-1(-/-) larvae, but this mutant shows 0% of complete lumen in L1 (Figure 1A). Wondering if ELT-2 expression is delayed in elt-7(-/-) end-1(-/-) mutants during embryogenesis that leads to the missed differentiation even if the expression seems normal in larvae.

In Figure 5L, each data point represents the average intensity of ELT-2::GFP in the gut cells. Although *elt-7(-) end-1(-)* double mutants contain fewer gut cells, the ELT-2 expression in these cells appears to be at normal levels in hatched larvae owing to the positive autoregulatory of ELT-2. Indeed, as the reviewer suggested, our computer model predicts that *elt-2* expression is severely delayed. Some cells lose expression entirely and do not appear to complete gut differentiation, while the majority express *elt-2* strongly in L1 larvae, presumably as a result of the positive feedback loop between ELT-7 and ELT-2. We have confirmed this prediction using a CRISPR-tagged mNeonGreen::ELT-2 reporter. These new results are included in the revised manuscript (Supplementary Figure 6; lines 315-320).

© 2022. Published by The Company of Biologists under the terms of the Creative Commons Attribution License (https://creativecommons.org/licenses/by/4.0/).

2. In Figure 6, 3 genes are involved in activation of terminal differentiation genes, which are END-1, ELT-7, and ELT-2. While in line 234-237 and Figure 1D and G, elt-7(-) end-3(-) mutants (with suboptimal expression of end-1), 61% of L1 has complete lumen. It seems that ELT-2 alone is not enough for the proper gut differentiation to have a complete lumen, while elt-7(-) end-3(-) mutant has a partial end-1 to help with the ELT-2 for terminal differentiation, probably explaining the higher ratio of complete lumen. However, end-1(ok558); elt-2(-) showed about 90% of complete lumen in L1 (Figure 1A), leading to a possibility that ELT-7 is the most important TF for terminal differentiation. However, this seems to contradict with the impression that ELT-2 is the most important TF for gut differentiation. Any thought on this observation.

We thank the reviewer for pointing out this important observation. Indeed, ELT-2 alone does not appear to be sufficient to drive robust gut differentiation and our data strongly argue against the role of ELT-2 as the "master regulator" of the endoderm lineage, in contrast to earlier claims in the literature. As we describe in the manuscript, *elt-2(-)* knockout animals produce an apparently complete, though defective organ and express most gut-specific genes. The essential function for ELT-2 in widespread gut morphological differentiation is revealed only when the strongly synergistic action of ELT-7 with ELT-2 is eliminated (Sommermann et al., 2010). It was found that ELT-7, when expressed under the control of the *end-1* and *elt-2* promotors can replace all other GATA factors in the GRN (Dineen et al., 2018). Additionally, our lab has shown that overexpression of ELT-7 causes widespread transdifferentiation of fully differentiated post-mitotic cells, showing that ELT-7 is a potent driver of intestinal differentiation (Riddle et al., 2013; Riddle et al., 2016). In this manuscript, we further found that END-1 may also drive differentiation in the absent of ELT-2 and -7. This extensive redundancy may be important to ensure reliable commitment to differentiation during rapid embryogenesis in *C. elegans*. We have discussed these important issues extensively in the revised manuscript (lines 611-623)

Reviewer 3 Advance Summary and Potential Significance to Field:

The authors describe the gene regulatory networks (GRN) that underlies the specification and differentiation of the endoderm, which gives rise to the intestine in C. elegans. The development of the C. elegans intestine is orchestrated through the activity of previously described GATA-type transcription factors, which are sequentially expressed and active. The authors combined different mutant backgrounds creating double / triple mutants and based on the observed mutant phenotypes they conclude that the transcription factors are acting in a recursive series of interlocked feedforward modules.

Overall, the manuscript is well written. The presented findings and derived conclusions can be considered as a refinement of many previously known aspects of C. elegans intestinal development.

Reviewer 3 Comments for the Author:

The authors describe in their manuscript 'Feedforward regulatory logic underlies robustness of the specification-to-differentiation transition and fidelity of terminal cell fate during C. elegans endoderm development' the gene regulatory networks (GRN) that underlies the specification and differentiation of the endoderm, which gives rise to the intestine in C. elegans. The development of the C. elegans intestine is orchestrated through the activity of previously described GATA-type transcription factors, which are sequentially expressed and active. The authors combined different mutant backgrounds creating double / triple mutants and based on the observed mutant phenotypes they conclude that the transcription factors are acting in a recursive series of interlocked feedforward modules.

Overall, the manuscript is well written. The presented findings and derived conclusions can be considered as a refinement of many previously known aspects of C. elegans intestinal development.

Major Concerns:
While the overall assessment of the feedforward loops is rather a refinement of previously described transcription factor actions that are relevant for gut development, some observations point towards new findings – yet the investigations are incomplete:

© 2022. Published by The Company of Biologists under the terms of the Creative Commons Attribution License (https://creativecommons.org/licenses/by/4.0/).

1. The observation that end-1 becomes depressed in early embryos when ELT-2/7 are depleted has not been described before. Unfortunately, the authors only argue that the effect is rather weak and provide no further experiments such using mutants to support the RNAi experiments (see also comment 3 below). This could have provided stronger effects.

We address this point in response to Reviewer 2, comment #3, as described above and added text to clearly acknowledge the potential caveats. We substantially tone down our claim of ELT- 2/7 repression of *end-1* expression in the revised manuscript.

2. Overall, the upregulation of pha-4 upon depletion of ELT-2 in the intestine is probably the most relevant finding in this study. The proposed 'dual role' of ELT-2 with regard to regulating pha-4 expression would be clear conceptual advancement. While ectopic pha-4 induction in the anterior gut in elt-7(-); elt-2(-) and elt-7(-) end-1(-); elt-2(-) mutants supports the transgene-based results for ectopic myo-2::gfp and ceh-22::gfp induction in the intestine, these effects need better experimental validation. All data concerning this important aspect are based on gfp-reporter transgenes! Such transgenes do not always reflect endogenous gene expression, in particular in modified genetic backgrounds. It is highly important to support the observations based on the gfp- reporter transgenes by assessing endogenous gene expression. smFISH is feasible and straightforward as this approach would not require generating new strains.

This issue is addressed as we describe in our response to comment #1 of reviewer 2. In the revised manuscript, we have included RNA-seq showing increased *pha-4* transcript abundance in *elt-2(-)* single and *elt-7(-); elt-2(-)* double mutants (new Supplementary Figure 16), which strengthens our conclusion. While smFISH can be an informative technique for examining transcriptional induction, we would assert that endogenously tagged gene reporters, as we have used throughout, are <u>much more relevant</u> for the goals and findings of this study as they provide a direct and biologically relevant readout of the critical parameter, <u>transcription factor abundance</u>, rather than <u>transcript abundance</u>, which may not reflect the amount of transcription factor present. To reiterate: it is the presence of a transcription factor, not its transcript, that underlies the architecture of the GRN and transcript analysis would not provide any important additional insights into the GRN structure beyond what we observe using a reporter of the gene products expressed from the endogenous loci.

For experiments showing derepression of pharynx genes, we found that *elt-7(-); elt-2(-)* double mutants and *elt-7(-) end-1(-); elt-2(-)* triple mutants show ectopic expression of *myo-2p::GFP* and *ceh-22p::GFP* – both expressed from multicopy transgenic arrays. We have further confirmed our findings with a third transgenic reporter in the revised manuscript (new Figure 4E). We argue that it is unlikely to observe the same modified genetic background and produce the same artifact in three independent transgenic strains. Additionally, the use of transcriptional reporters eliminates any concerns about overexpression effects.

3. It is not clear why in some cases elt-2(-) combined with elt-7 RNAi is being used (e.g. when scoring opt-2p::mCherry) although in several other cases (e.g. when scoring myo-2p::GFP) the double mutant elt-2(-); elt-7(-) has been used. This is somewhat inconsistent and using mutant alleles to support RNAi results should be used whenever possible.

We note that confirming the efficacy of RNAi is particularly critical when <u>negative</u> results are observed and we have observed only <u>positive</u> effects in all the reported RNAi experiments. We have obtained strong evidence that *elt-7* RNAi is highly penetrant and is therefore appropriate for epistasis analysis. For example, the results with the chromosomal *elt-7(-)*; *elt-2(-)* double mutants, and those obtained by RNAi of *elt-7* in the *elt-2(-)* mutant are indistinguishable, with sporadic, all-or-none, block to gut differentiation along the length of the animals (Supplementary Figure 7). Further evidence of *elt-7* RNAi penetrance can be found in Figures 2J, 3, and 4I, as well as the new Supplementary Figures 6 and 10 and Supplementary Figures 11, and 15.

Minor concerns:

4. Knowing that the authors are experts and experienced with identifying in a proper way intestinal tissue it would be good to provide some evidence that the ectopic signals for myo-2, ceh-22, pha- 4 are indeed in intestinal cells.

© 2022. Published by The Company of Biologists under the terms of the Creative Commons Attribution License (https://creativecommons.org/licenses/by/4.0/).

We agree that it would indeed be interesting to further investigate the fates of the endodermal descendants at higher resolution; however, the location of these signals suggest they are included in endoderm-derived cells. Given the exhaustive amount of experimental data already included in the revised manuscript and the focus of the study, we feel that the additional experiments (for example, performing cell lineage analysis) is beyond the scope of the paper.

5. *The title is too long!*

We have now shortened the title to "*Feedforward regulatory logic controls the specification-to-differentiation transition and terminal cell fate during* C. elegans *endoderm development.*"

6. *line 84 (page5): '…to a clone 20 cells…' – seems like 'of' is missing*

Corrected.

7. *Authors provide no idea how repression of end-1 by ELT-2/-7 could take place at the molecular level.*

While it would be of interest to study how *end-1* is regulated by ELT-2 and -7, ChIP-seq data are not available for the GATA factors in this study at the necessary early embryonic stages (http://epic.gs.washington.edu/modERN/). In this manuscript, we have reported preliminary findings that *end-1* is downregulated in *elt-2/7(RNAi)* mutants. Investigating the molecular mechanisms require a new study that is well beyond the scope of the current paper, which, again, already contains very extensive data and support.

Second decision letter

MS ID#: DEVELOP/2021/200337

MS TITLE: Feedforward regulatory logic controls the specification-to-differentiation transition and terminal cell fate during C. elegans endoderm development

AUTHORS: Chee Kiang Ewe, Erica M Sommermann, Josh Kenchel, Sagen E Flowers, Morris F Maduro, Pradeep M Joshi, and Joel H Rothman

I have now received all the referees reports on the above manuscript, and have reached a decision. The overall evaluation is positive and Development would be interested in publishing the manuscript. However, as reviewer 2 suggests, it would be important to explain why the removal of the GFP::MED-1 and MED-2::GFP data does not impact the findings before we can proceed further. Please attend to all of the reviewers' comments in your revised manuscript and detail them in your point-by-point response. I look forward to receiving your revised manuscript.

Reviewer 1

Advance summary and potential significance to field

The authors have substantially strengthened their manuscript by adding new data, analysis, and key clarifications previously missing. Importantly, the computational model is now well integrated to support the main findings of the study. I believe the revised manuscript considerably increases our understanding of the endoderm GRN and will be of interest to scientists modelling other TF-based GRNs, and as such it is suitable for publication in Development.

Comments for the author

N/A

© 2022. Published by The Company of Biologists under the terms of the Creative Commons Attribution License (https://creativecommons.org/licenses/by/4.0/).

Reviewer 2

Advance summary and potential significance to field

This manuscript aimed to decipher the functional redundancies and interactions between the GATA transcription factors for faithful gut development in C.
elegans. Using a combination of single/double/triple genetic mutants or RNAi they found that removal of a single factor or alternate factors in the cascade results in a much milder effect on endoderm development and gut differentiation than elimination of any two factors that are sequentially expressed factors within the regulatory cascade, which appears to be upheld by the modeling analysis. Consequently, they propose a feedforward regulatory logic for the gut development. They further claimed that END-1 mediates gut specification-to-differentiation. They finally showed the roles for key GATA factors in establishing spatial regulatory state domains by acting as transcriptional repressors that appear to define the boundaries of the digestive tract. The genetic work has been beautifully done. The regulatory control by GRN is definitely an exciting area that is worthy of attention.

Comments for the author

The authors have addressed most of my concerns/confusions in the revised manuscript. Well done! Here a few more comments.

1. In response to my first comments, they claimed “we have removed the analysis of GFP::MED-1 and MED-2::GFP fusion proteins from the revised manuscript as a result of the concerns about integrated arrays. This change has no impact on our major conclusions.” Can you clarify why removal of such information has no impact on your major concussions, which still claim sequential relationship between the two factors?
2. Figure 5 mask the title of its legend.

Reviewer 3

Advance summary and potential significance to field

My comments and concerns based on the original submission have been sufficiently addressed in the revised version. The authors provided additional information such as clarifying that endogenous reporters have been used rather than transgenes.

Comments for the author

I have no further comments.

Second revision

Author response to reviewers' comments

We thank you and the reviewers for the thoughtful comments on our manuscript. We have now addressed the final comments from the reviewers, which are indicated in italics. Our responses follow each comment.

Reviewer 1

The authors have substantially strengthened their manuscript by adding new data, analysis, and key clarifications previously missing. Importantly, the computational model is now well integrated to support the main findings of the study. I believe the revised manuscript considerably increases our understanding of the endoderm GRN and will be of interest to scientists modelling other TF- based GRNs, and as such it is suitable for publication in Development.

© 2022. Published by The Company of Biologists under the terms of the Creative Commons Attribution License (https://creativecommons.org/licenses/by/4.0/).

We are grateful to this reviewer for their supportive comments and recommendation for publication.

Reviewer 2

This manuscript aimed to decipher the functional redundancies and interactions between the GATA transcription factors for faithful gut development in C. elegans. Using a combination of single/double/triple genetic mutants or RNAi, they found that removal of a single factor or alternate factors in the cascade results in a much milder effect on endoderm development and gut differentiation than elimination of any two factors that are sequentially expressed factors within the regulatory cascade, which appears to be upheld by the modeling analysis. Consequently, they propose a feedforward regulatory logic for the gut development. They further claimed that END- 1 mediates gut specification-to-differentiation. They finally showed the roles for key GATA factors in establishing spatial regulatory state domains by acting as transcriptional repressors that appear to define the boundaries of the digestive tract. The genetic work has been beautifully done. The regulatory control by GRN is definitely an exciting area that is worthy of attention.

The authors have addressed most of my concerns/confusions in the revised manuscript. Well done! Here a few more comments.

1. In response to my first comments, they claimed “we have removed the analysis of GFP::MED- 1 and MED-2::GFP fusion proteins from the revised manuscript as a result of the concerns about integrated arrays. This change has no impact on our major conclusions.” Can you clarify why removal of such information has no impact on your major concussions, which still claim sequential relationship between the two factors?

We thank the reviewer for their supportive comments. Although single-cell transcriptomic data and our analysis of transgene expression strongly suggest that med-2 is activated slightly early than med-1, examining their endogenous expression would be required to confirm the evidence from analysis of the transgenes, as pointed out by the reviewer. The reagents to perform those experiments are not available and would require an extensive effort to generate. Hence, in response to the reviewer’s earlier comment, we have removed the analysis of GFP::MED-1 and MED-2::GFP fusion proteins and substantially toned down the claim that the two genes are differentially regulated (although we believe the evidence is reasonable). Nonetheless, the sequential relationship between MED-2 and MED-1 is, in fact, strongly supported by our genetic data showing that med-1(-); end-3(-) double mutants exhibit a much more severe developmental defects than med-2(-); end-3(-) (Figure 1C; lines 215-227). Furthermore, it was previously shown that embryos lacking MED-1 show a weaker loss-of-gut phenotype than those lacking MED-2 when SKN-1 function is also debilitated (Maduro et al., 2007) (see Discussion lines 421-426), strongly supporting the model that MED-2 acts upstream of MED-1 in the endoderm GRN.

2. Figure 5 mask the title of its legend.

Corrected.

Reviewer 3

My comments and concerns based on the original submission have been sufficiently addressed in the revised version. The authors provided additional information such as clarifying that endogenous reporters have been used rather than transgenes.

I have no further comments.

We are grateful to the reviewer for their supportive response.

© 2022. Published by The Company of Biologists under the terms of the Creative Commons Attribution License (https://creativecommons.org/licenses/by/4.0/).

Third decision letter

MS ID#: DEVELOP/2021/200337

MS TITLE: Feedforward regulatory logic controls the specification-to-differentiation transition and terminal cell fate during C. elegans endoderm development

AUTHORS: Chee Kiang Ewe, Erica M Sommermann, Josh Kenchel, Sagen E Flowers, Morris F Maduro, Pradeep M Joshi, and Joel H Rothman
ARTICLE TYPE: Research Article

I am happy to tell you that your manuscript has been accepted for publication in Development, pending our standard ethics checks.

© 2022. Published by The Company of Biologists under the terms of the Creative Commons Attribution License (https://creativecommons.org/licenses/by/4.0/).
